# Civilian national service programs can powerfully increase youth voter turnout

**DOI:** 10.1073/pnas.2122996119

**Published:** 2022-07-11

**Authors:** Cecilia Hyunjung Mo, John B. Holbein, Elizabeth Mitchell Elder

**Affiliations:** ^a^Department of Political Science, University of California, Berkeley, CA 94720;; ^b^Frank Batten School of Leadership and Public Policy, University of Virginia, Charlottesville, VA 22903;; ^c^Center for the Study of Democratic Politics, Princeton University, Princeton, NJ 08544;; ^d^Bobst Center for Peace and Justice, Princeton University, Princeton, NJ 08544

**Keywords:** national service programs, voter turnout, regression discontinuity, political socialization, Teach For America

## Abstract

Low rates of youth voting are a feature of contemporary democracies the world over, with the United States having some of the lowest youth turnout rates in the world. However, far too little is known about how to address the dismal rates of youth voter participation found in many advanced democracies. In this paper, we examine the causal effect of a potentially scalable solution that has attracted renewed interest today: voluntary national service programs targeted at the youth civilian population. Leveraging the large pool of young people who apply each year to participate in the Teach For America (TFA) program—a prominent voluntary national service organization in the United States that integrates college graduates into teaching roles in low-income communities for 2 y—we examine the effect of service participation on voter turnout. To do so, we match TFA administrative records to large-scale nationwide voter files and employ a fuzzy regression discontinuity design around the recommended admittance cutoff for the TFA program. We find that serving as a teacher in the Teach For America national service program has a large effect on civic participation—substantially increasing voter turnout rates among applicants admitted to the program. This effect is noticeably larger than that of previous efforts to increase youth turnout. Our results suggest that civilian national service programs targeted at young people have great promise in helping to narrow the stubborn and enduring political engagement gap between younger and older citizens.

## Significance

Enrolling young people to participate as Teach For America (TFA) teachers has a large positive effect on rates of voter turnout among those young people who participate. This effect is considerably larger than many previous efforts to increase youth voter turnout. Each year, TFA places thousands of young adults in 2-y teaching positions in disadvantaged communities around the United States. After their 2 y of service, we find that these young adults vote at a rate 5.7 to 8.6 percentage points higher than that of similar nonparticipant counterparts. Our results suggest that civilian national service programs targeted at young people show great promise in narrowing the enduring participation gap between younger and older citizens in the United States.

**Fig. 1.**
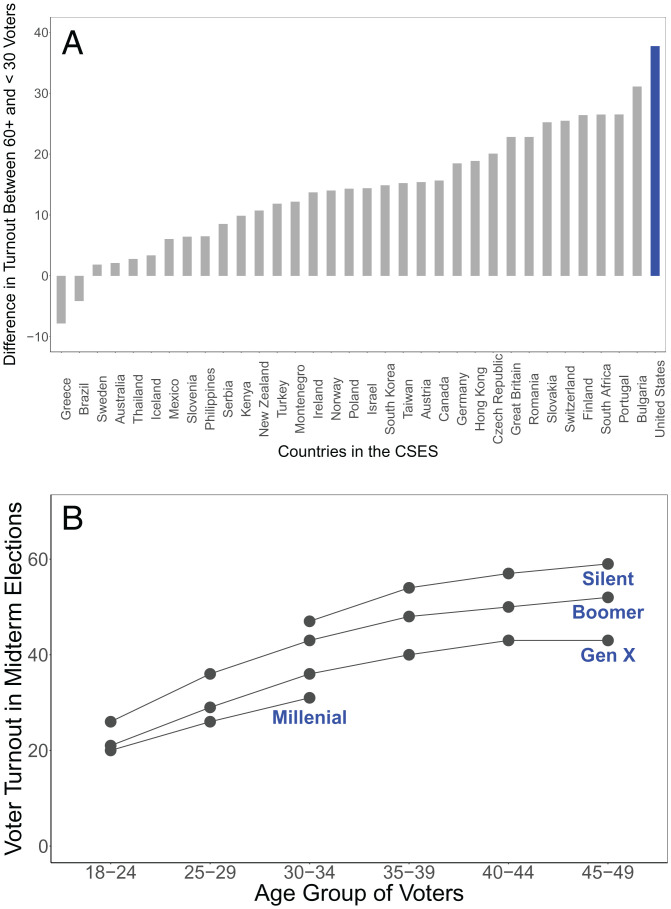
Youth voter turnout in the United States is low and may be declining. (*A*) The age gap in voter turnout across 34 countries in the Comparative Study of Electoral Systems (Module 4; via ref. 5). Bars show the turnout rate for those 60+ y old minus those 18 to 29 y old in each country. (*B*) Voter turnout in the United States (1978 to 2014 midterms) by age and generation. Source*:* Current Population Survey November Supplement (recreated as reported by Pew Research Center and in ref. 5). Following Pew’s coding, Millennials are defined as those born between 1981 and 1996, Generation X as those born between 1965 and 1980, Baby Boomers as those born between 1946 and 1964, and the Silent Generation as those born between 1928 and 1945.

Political engagement is one of the central pillars of democracy (1–4). Yet, the United States holds the dubious honor of having one of the lowest and most unequal rates of political participation in the world. One of the largest gaps in voter turnout is by age—in the United States, it is not altogether uncommon to see older citizens turn out at a rate twice as high as that of their younger counterparts (5). Fig. 1 puts this gap into perspective. Fig. 1*A* shows the gap between older (i.e., those ages 60+) and younger (i.e., those ages 18 to 29) voters across the world; Fig. 1*B* shows how various generations in the United States have aged into voting. As can be seen, the United States has the largest gap between older and younger voters in measured countries, and there is some evidence that youth turnout may be getting lower over time. Based on data from the Current Population Survey, young voters are turning out at rates lower than previous generations did at the same age. Whatever comparison point one uses, however, youth voter turnout in the United States is, in a word, dismal. This pattern has many known causes—including the uniquely high obstacles to registration and voting in the United States and other societal inequities (5–16).

However, there exists far too little knowledge of what can be done—particularly at scale—to ameliorate this large, consequential, and stubborn gap in political participation. At present, we know that many campaign-based efforts to get out the vote have very modest effects (17–20). While some get-out-the-vote programs have shown promise at raising youth turnout (e.g., ref. 6), these interventions are few and far between and are often implemented only in select areas. In this paper, we take a step toward filling this lacuna in the scientific literature by exploring one of the core proposed public policies designed to help raise youth civic engagement: voluntary civilian (i.e., nonmilitary) national service programs, which we simply refer to as national service programs hereafter. We use an established and prominent national service program—Teach For America (TFA)—as our empirical case.

Given the core role that young people play in shaping the future health of civic life, young people have been the objects of many service-based efforts to inculcate the values and practices upon which democratic citizenship depends (21). When discussing national service programs, scholars have examined two types of programs—those voluntary and those nonvoluntary. Voluntary national service programs, like the Peace Corps and AmeriCorps, stand in contrast to compulsory national service programs, like those involving mandatory military service (22–24), in both the nature of their service activities and their noncompulsory element. Many voluntary national service programs have historically targeted and attracted young adults. For example, the National Civilian Community Corps (NCCC), an AmeriCorps program, requires that participants be between 18 and 26 y old, and about 85% of Peace Corps volunteers have historically been recent college graduates (25).

Numerous scholars have found evidence of a positive relationship between voluntary youth national service and future civic engagement. Indeed, there are many theoretical mechanisms by which national service may affect participation rates (for a full list, see *SI Appendix*, section B.1). Extant sociology research finds that participation in youth service is associated with increases in 1) feelings of civic duty (26); 2) understanding of civic issues facing a community, as well as feelings of obligation and connection to a community (27, 28); 3) engagement in civic life (e.g., participating in a community action program, volunteering, working for a nonprofit, or participating in a community service organization) (29–31); and 4) a sense of duty to improve government systems for the common good (28, 32).

Despite these promising findings from conditional-on-observable research designs, we currently lack large-scale studies that are designed to detect the causal effects of national service programs. This raises serious inferential concerns, as previous positive associations may be driven more by the types of people who select into national service programs rather than the independent effects of national service programs. Moreover, while some evidence indicates that service learning encourages participants to better understand the challenges communities face (33), some scholars have noted that there is less evidence on the impact of service learning on political behavior such as voting or interacting with government officials (34). More pointedly, one prominent study of a youth national service program recently argued that graduates of that program are engaged in fewer service activities/civic duties, vote at lower rates, and are employed in less “prosocial” jobs than an observationally similar comparison group (21). However, these less-positive studies did not examine validated voter turnout, nor did they identify the causal effect of participating in a civilian voluntary national service program.

To start to address these inferential gaps in studies of the effects of national service programs on civic engagement and political participation, we examine the causal effect of participating in TFA, a prominent national service program established in 1990 with a mission “to enlist, develop, and mobilize as many as possible of our nation’s most promising future leaders to grow and strengthen the movement for educational equity and excellence.”* To accomplish this goal, TFA places college graduates into teaching positions in low-income and underserved communities throughout the United States. Applicants who are admitted and matriculate into the program serve as teachers for a period of 2 y.^†^ Alumni may, and often do, continue to serve as teachers at their assigned schools; however, active service time in TFA is limited to 2 y.

To evaluate the effect of this program, we utilize a large-scale, proprietary administrative dataset from TFA that contains information on all program applicants during the 2007 to 2015 period. Because nearly 80% of TFA applicants from this period are younger than 25 y old, this represents a unique opportunity to study the causal effects of a national service program on youth turnout. We match these TFA data with nationwide voter file data that contain information on all registered voters (N ≈ 200 million) in the United States. With this combined dataset, we take advantage of a natural experiment wherein we compare individuals who were marginally recommended to be accepted to participate (based on TFA’s proprietary admittance threshold) to those who were marginally recommended to be rejected from participating. This threshold allows us to employ a fuzzy regression discontinuity design, a method of causal inference developed and refined over the past 60 y that leverages continuity in potential outcomes around an arbitrary cutoff (35–40) and benchmarks well with randomized control trial estimates (41).

We find that participating in TFA has a large effect on youth political participation. Ceteris paribus, 2 y after applying for TFA (which is the duration of the TFA program), individuals who scored marginally above the admissions cutoff score and, hence, were recommended for admission into TFA, vote at a rate 5.7 to 8.6 percentage points higher than those who were marginally rejected (conventionally this estimate is called the intention to treat [ITT] effect). When we account for the fact that the admissions cutoff score has a probabilistic (rather than deterministic) relationship to likelihood of selection into the program, we estimate that participants in TFA are 30.1 to 42.3 percentage points more likely to vote than similar nonparticipants (conventionally this is called the complier average causal effect [CACE]). To put these estimates into perspective, the ITT effect is 3 to 14 times larger than standard get-out-the-vote (GOTV) programs and ∼20 to 30% of the entire turnout gap between old and young voters (which is about 30 percentage points, depending on the election considered). Regardless of the comparison point one uses, these effects are large.

Our work has important practical and conceptual implications. The House and the Senate are currently considering whether to expand national service to bolster COVID-19 pandemic recovery (see, for example, S.3964 and H.R.1162), and the expansion of national service is regularly discussed in Congress (for the full list of the over 250 bills having to deal with national service in recent Congresses, see *SI Appendix*, Table S2). States are also investing in regional service programs targeting youth. For example, in January 2022, the state of California announced the creation of the #CaliforniansForAll College Corps to create debt-free college pathways for students who commit to serve their communities (42). While future studies of the causal effect of different types of national and regional service programs are needed, our findings provide insight into the potential benefits of voluntary civilian service programs targeting youth. We find that the TFA experience catalyzes young participants to vote in a way rarely seen in previous studies. More broadly, these results help us understand how exposure to various social ills, paired with the act of serving to meet critical needs in disadvantaged communities, can affect the prosocial behavior of youth in American society. Voluntary civilian service experiences have the potential to change how youth act in the democratic domain and, in so doing, can help narrow the stubborn participatory gap between young and older citizens.

## Results

To estimate the effect of TFA admittance and participation on voter turnout, we linked a national voter file database from the Data Trust LLC (snapshot taken 29 September 2017) to records from TFA’s administrative data for the 2007 through 2015 admissions cycles and a 2015 to 2016 original survey of TFA applicants. TFA’s administrative data include an application quality score that strongly and discontinuously predicts admission into the program. We employ a regression discontinuity design around the TFA admittance cutoff to estimate the effect of admission to and participation in TFA on voting in subsequent national elections. (We describe the TFA admissions cutoff in greater detail in *Materials and Methods* and *SI Appendix*, sections A.3 and A.4. There too, we describe the methods we used to link TFA and voter file records.)

**Fig. 2.**
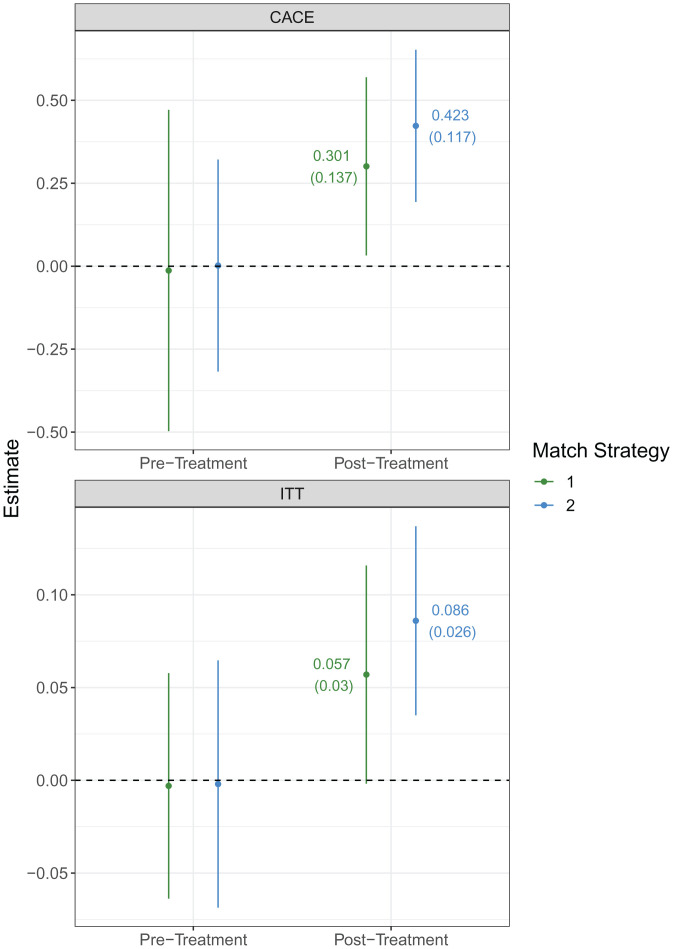
Effect of TFA experience on voter turnout. (*Top*) The CACE of TFA acceptance on turnout. (*Bottom*) The ITT effect. Each panel shows the effect of the treatment on turnout before the treatment occurred, followed by the effect of treatment on turnout in elections 2 or more y after treatment.

Fig. 2 displays our regression discontinuity estimates, where the outcome of interest is whether one voted in either the 2012 or the 2014 election (see *SI Appendix*, section A.7 for an alternative presentation of these results). The green lines in Fig. 2 reflect a matching strategy, hereafter referred to as “Match 1,” which employs names from TFA’s application file and year of birth from the survey. The blue lines reflect an alternative matching strategy, hereafter referred to as “Match 2,” employing names and year of birth from TFA’s administrative file. In the case of Match 2, when year of birth was missing, we employed applicants’ graduation year to estimate their year of birth (see *SI Appendix*, section A.4 for more details). Overall, these matching strategies lead us to a similar conclusion and illustrate the robustness of our results.

In the left pair of estimates in each panel of Fig. 2 (see also *SI Appendix*, Fig. S6 *A* and *B* for regression discontinuity design [RDD] plots), we compare rates of voter turnout before TFA applicants were either admitted to or rejected by the program across the admissions cutoff. As can be seen, before applying to the TFA program, eventual marginal admits and marginal declines voted at rates that are statistically and substantively indistinguishable from one another. This can be seen by the insignificant and close-to-zero estimates of the CACE and ITT effect in the pretreatment period. In these pretreatment elections, the CACE ranges from –1.3 percentage points (*P* = 0.96) to 0.2 percentage points (*P* = 0.99), and the ITT effect ranges from –0.3 (*P* = 0.93) to –0.2 percentage points (*P* = 0.96). In other words, TFA participants were statistically equally as likely to participate in elections as nonparticipants when they applied to join—and before they had completed any parts of—the TFA program. This pretreatment balance lends support to the key assumption that barely admitted and barely rejected applicants would not have voted at different rates at the cutoff without the intervention of TFA.

In contrast, the second pair of estimates in each panel of Fig. 2 (see *SI Appendix*, Fig. S6 *C* and *D* for RDD plots) shows the rates of voter turnout for TFA applicants 2 or more y after their admittance into or rejection from the program (i.e., when a participant would have completed their TFA participation). We find that TFA participation increases voter turnout in the 2012 and 2014 elections for treated respondents. The intention to treat (Fig. 2, *Bottom*) estimate ranges from 5.7 (*P* = 0.057) to 8.6 percentage points (*P* < 0.001). The complier average causal effect (Fig. 2, *Top*) of TFA participation ranges from 30.1 (*P* = 0.028) to 42.3 percentage points (*P* < 0.001). If we examine whether TFA participation has a positive effect on the proportion of elections in which an applicant voted, we similarly find positive effects (see *SI Appendix*, Fig. S9, and the rest of *SI Appendix*, section A.9 for other alternative dependent variables).

These estimated effects are substantively meaningful. They are large, but not unreasonable given the nature of the program. In considering the magnitude of our effects, we pause a moment here to note the size and scope of the TFA program and TFA’s known effects on the antecedents to voting. Unlike many get-out-the-vote programs, which draw the attention of voters for a matter of minutes (at most), the TFA experience is quite an intensive treatment. TFA is not a light nudge, but is, rather, a fully immersive 2-y treatment. Those who serve as TFA teachers spend all of their working—and many of their nonworking—hours being exposed to a whole new set of experiences, networks, and cultures. In this immersive environment, TFA teachers are exposed to many students, teachers, administrators, parents, communities, and/or contexts that are outside of their normal realm of experience. Previous research on the attitudinal effects of TFA has shown that this immersive experience fundamentally shifts some of the antecedents to voting. For example, past research on TFA shows that TFA substantially increases participants’ dissatisfaction with the current political system, while simultaneously increasing their ability to see the plight of disadvantaged communities and empowering them with the optimism and efficacy needed to believe that positive reform in the policy arena is possible (33, 43). Unlike other get-out-the-vote programs (44, 45), TFA fundamentally transforms treated subjects’ attitudes. In short, we have reason to expect that the effects we observe will be larger than those of many of the lighter-touch nudges to vote studied in the past. And this is, in fact, exactly what we observe.

There are several benchmarks against which we can compare our effect sizes. None of these are perfect—each comes with its own limitations and assumptions—but using them together, we can get a sense of the magnitude of our effect. Moreover, all end up pointing in the same direction—that the effect of TFA is large and meaningful. Below, we mostly benchmark our ITT effects to the ITT effects obtained from other comparable interventions intended to increase voter turnout, to make the comparisons as equivalent as possible.

First, we can compare the effect of TFA participation to the voter turnout gap between young and older voters. As Holbein and Hillygus (5) report, in the two most recent national elections, the gap between young (i.e., those ages 18 to 29) and older (i.e., those ages 60+) is somewhere between 28.0 (in 2016) and 32.9 (in 2018) percentage points. As such, the ITT effect of TFA on voter turnout represents 17.3 to 30.7% of the entire gap between younger and older voters (depending on which of the two estimates of the effect or which turnout gap estimate is used). This suggests that an effect of the size of TFA’s on all program applicants—if translated to youth more generally—would not completely close the gap between young and older voters, but it would take an important step toward doing so. Moreover, if we consider the effect size local to compliers (i.e., the CACE) instead and apply this to the broader youth population, then TFA would be sufficient to effectively close the gap between younger and older voters. (We discuss whether generalizing our effects to a broader population is wise in the *Discussion.*)

Second, we can compare our effects to other more immersive education-based treatments. We first compare our effects to estimates of the average effects of civics education (46–48). Holbein and Hillygus (5) report that the average effect of implementing Advanced Placement (AP) Government courses on turnout ranges from 0.5 to 3.1 percentage points and the average effect for implementing AP US History courses ranges from 1.9 to 2.5 percentage points. Our effects are somewhere between 1.8 and 17.2 times as large as the effects of these commonly taken courses that are often advocated as a means of increasing youth engagement. Our effect also compares favorably to those of more tailored pilot civics education programs. The First-Time Voter program, which uses in-classroom voting and registration tutorials, increases youth turnout by 5.7 percentage points (ITT effect) on average (5, 6). Our ITT effects benchmark well with those from this program—being right in line or somewhat larger. Our effects are also right on par with the lottery-based ITT estimate of Democracy Prep Charter Schools, which increase youth turnout by 7.2 percentage points (5, 49). In short, TFA appears to be much more effective than the average standard civics curricula and right in line with (if not, in some cases, slightly larger than) other educational programs of similar duration and/or intensity.

**Fig. 3.**
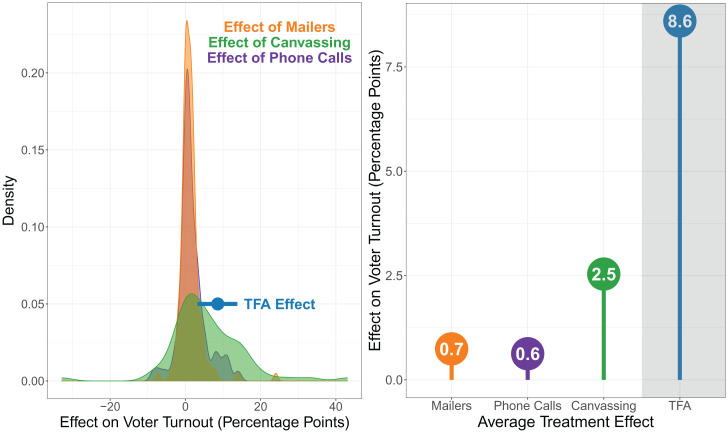
The effect of TFA experience relative to other GOTV interventions. Data from GOTV interventions come from 75 phone-banking experiments, 147 mailer experiments, and 73 canvassing experiments included in Green, McGrath, and Aronow (51). (*Left*) The effect of TFA (match 2) as a coefficient with corresponding 95% CIs next to the distributions of the GOTV effects. (*Right*) A lollipop chart that places the average effect of TFA relative to the average effect of each GOTV treatment.

Finally, we can compare our effects to other commonly used strategies to get out the vote (i.e., GOTV)—such as phone calls, canvassing, and mailers. Although these GOTV interventions are clearly not the same as a national-service–based TFA treatment—being significantly smaller in their treatment intensity than the TFA program—GOTV efforts are the most widely studied approach to increase voter turnout and the one approach that most frequently uses methods for causal inference (50). To benchmark our effects to GOTV interventions, we draw on a recent meta-analysis conducted by Green, McGrath, and Aronow (51) that pulled together effect estimates from 75 phone-banking experiments, 147 mailer experiments, and 73 canvassing experiments. Fig. 3 plots our Match 2 effect size relative to these GOTV experiments. The effect of TFA is 14.3 times larger than that of the average phone-banking GOTV intervention, 12.3 times larger than that of the average mailer GOTV intervention, and 3.4 times larger than that of the average canvassing GOTV intervention. (The comparable numbers for Match 1 are 9.5 times larger, 8.1 times larger, and 2.3 times larger, respectively.) Compared to the whole distribution of GOTV effects, the average TFA effect falls at the 93rd percentile of phone-banking interventions, the 69th percentile of canvassing interventions, and the 98th percentile of mailer interventions. Even if we cherry pick the most effective youth canvassing approach found in Green, McGrath, and Aronow’s dataset (51)—which finds a CACE of about 22 percentage points, on average—our effects are still noticeable, being about 1.4 to 1.9 times larger than in-person contact treatment effects.

In short, all comparison points suggest that TFA’s effect on voter turnout is large and meaningful. Indeed, national service experience through TFA likely does not completely close the gap between young and older voters, but it does make a meaningful step toward doing so. In interpreting the magnitude of these effects, however, one important point about treatment scope and exposure is worth reiterating. TFA is a 2-y program, whereas many previous voting treatments are much shorter. The effects that we observe are wholly consistent with larger, more immersive programs having larger effects on voter turnout.

Our effects are robust to various alternate specifications. For example, when we use the admissions score as an instrument for TFA program completion rather than matriculation, we see that the effects are stronger (*SI Appendix*, Fig. S14). Further, while we prioritize the 2012 and 2014 elections given that those elections are closest to when participants were surveyed to get reliable information on their state of residence, if we expand the analysis to include the 2008 and 2010 elections, results largely remain the same (*SI Appendix*, Figs. S12 and S13). Moreover, we note that one may be concerned that effect sizes are overestimates if nonadmits are pursuing work that may socialize individuals to participate less in politics. However, they could also be underestimates if nonparticipants systematically worked in sectors that may also encourage greater political participation. To explore this question, we examine the job sectors of nonparticipants since 2007, the first cohort year in our study. As seen in *SI Appendix*, Figs. S15–S17, over one-third of nonparticipants pursued work in the education sector. The next two most-represented sectors are the nonprofit and legal sectors. Nearly half of nonparticipants entered the legal, nonprofit, and education sectors, and there are no documented reasons that these three sectors would depress voter turnout. As such, it is unlikely that we overestimate the effect of TFA due to the career trajectory of nonparticipants.

When it comes to treatment effect heterogeneity, the effect of TFA is similar for Whites and non-Whites and across several geographic areas in the United States, but effects appear to be larger among men than women and among those with higher rather than lower socioeconomic status, as measured by federal Pell Grant status (*SI Appendix*, Fig. S7). However, as with most tests of treatment effect heterogeneity, statistical power may be an issue here. Moreover, we do not see consistent evidence that the positive effects we detect are present after receiving a smaller “dose” of the program (i.e., receiving only a few months or 1 y of the program), although we interpret these results with caution given the reduced sample size (*SI Appendix*, Figs. S10 and S11).

We consider potential mechanisms in *SI Appendix*, section B.1. We note here that mechanism testing is inherently difficult as observing all potential mechanisms is difficult, if not impossible (52). We note (briefly) here one mechanism that is unlikely to be driving our results—direct mobilization of TFA participants by the TFA organization itself. Explicit efforts from the national service organization to increase political participation are not a likely mechanism for the effects we observe, as the Serve America Act explicitly prohibits national service programs from doing so (53) and, as such, no mobilization/registration programs occur under the umbrella of the TFA organization. That said, it is possible that while enrolled in TFA, participants are exposed to mobilization from their peers and organizations that oversee and interact with teachers. The shift in the network with which TFA participants interact likely plays some role in the effects we see, along with the fundamental attitudinal transformation that follows participating in TFA (33, 43). While the mechanisms driving the effects we observe are likely many and complex—and worthy of examination in future research—this much is clear: For many young people, participating in TFA substantially increases the chances that they will be active in the democratic process.

## Discussion

Participating in Teach For America—as thousands of young people across the United States do each year—substantially increases the odds that these young people will participate in politics. Pairing comprehensive records of those who have applied to TFA with a unique nationwide voter file, we have shown compelling causal evidence that serving as a TFA teacher increases voter participation substantially. Individuals who participate in TFA vote at a rate 5.7 to 8.6 percentage points higher than those who do not. Regardless of the comparison used to benchmark these estimates, this effect is large—suggesting that national service programs like TFA have the potential to fundamentally change the civic engagement patterns of those who choose to participate. This is significant, as a democracy requires citizens that actively participate in public deliberation rather than citizens that are apathetic, alienated from the political process, and withdrawn into the private sphere of family, career, and personal pursuits (54).

Our work uses a natural experiment unique in its scale and size to study the causal effect of national service participation on youth voter turnout; however, it should not be the last to examine this important topic. Future research is needed to explore questions of external validity. While we established that the effects of TFA participation are positive across many subsets of the population of applicants, we cannot estimate how effects would differ in other populations. This may be especially important, as individuals who apply to national service programs may differ from the general population in important ways. Future work should also explore whether effects like those we document here generalize to other subsets of compliers, like to noncollege graduates and to older citizens (to name a few). That said, scholars doing such work in the future would do well to note that, to a certain extent, all individual voluntary youth service programs—given their voluntary nature—likely experience selection effects into application. Conversely, while mandatory national service programs may cover a broader subset of participants—being mandatory, after all—they may struggle to produce all-else-equal counterfactual comparisons. Moreover, mandatory national service programs may have different effects on participation given differences in the potential attitudinal effects of compelling service. Future research would also do well to see whether we observe similar causal effects of other voluntary service programs beyond TFA, like the Peace Corps. Doing so would allow us to answer questions like whether the effects we document are larger for national service programs that involve teaching, as there is suggestive evidence that under certain conditions, being a teacher increases voter turnout (55) (see *SI Appendix*, section B.1 for a discussion of how the act of teaching is unlikely to be driving the entirety of our effect). Simply put, the magnitude of effects may differ by the type of service one is charged to provide.

In 1910, US philosopher William James argued that youth service could be a mechanism by which “a stable system of morals of civic honor builds itself up” (ref. 56, p. 24). And since President John F. Kennedy famously challenged Americans—“Ask not what your country can do for you, ask what you can do for your country” (57)—national service programs have multiplied, with over 1.3 million Americans answering Kennedy’s call to serve (25, 58). However, 1.3 million is a small fraction of the US adult population eligible to apply to such programs. Currently, an expansion of national service is being proposed by the House of Representatives and the Senate—via the CORPS Act (see, for example, S.3964 and H.R.1162; for the full list of over 250 bills having to deal with national service over the past 13 Congresses, see *SI Appendix*, Table S2)—as a necessary response to bolster COVID-19 recovery. The intention of these efforts is to create more opportunities for young people to help the nation combat, and recover from, the devastation of the COVID-19 pandemic (59). In the midst of the pandemic, recognizing how polarized Americans are today, the Editorial Board of the New York Times recently wrote that young Americans should be required to give a year of service in response to a “bitterly divided America struggling with a pandemic ” (60). Moreover, campaigns like Serve America Together have been initiated to make 1 y of full-time public service a common expectation of and opportunity for all young Americans. Our results speak to the promise of these efforts.

We find that TFA, which explicitly aims “to improve lives, strengthen communities, and foster civic engagement through service and volunteering” (61), meaningfully increases civic participation. These results are vitally important given the low rates of civic participation in the United States. Young people in the United States have some of the, if not the, lowest rates of civic engagement in the world. Moreover, looking at voter turnout by age and generation, we see that each generation is voting less than its previous generation at the same age. Our results suggest that service interventions, like TFA, which targets youth for a voluntary national service experience, have great potential to increase the chances that citizens participate in politics.

## Materials and Methods

Our analysis leverages administrative data from TFA, a nationwide voter file, and a survey of TFA applicants. The Committee for Protection of Human Subjects (CPHS) at University of California, Berkeley provided institutional review board approval to conduct this research (2020-02-13026). TFA maintains detailed selection data (e.g., name, date of birth, telephone number, current address, undergraduate university, application year, selection score, admissions decision, matriculation decision, and demographic characteristics) at the time a given applicant applied to the program, and we use this information for all applicants who were competitive to join the program. We focus on the set of applicants who made it to the final round of interviews for the 2007 to 2015 application cycles and, hence, received a selection score. This amounts to 120,329 applicants. All of these applicants were also targeted by Mo and Conn (33) between 1 October 2015 and 31 March 2016 to complete an online survey. Over one-quarter of the targeted applicants (27.1%; American Association for Public Opinion Research R2 response rate) responded to the survey, which importantly asked about their current state of residence. After removing noncitizens and individuals who did not provide a current state of residence, we were left with a sample of 28,662 potential voters (see *SI Appendix*, Table S1 for survey sample demographic characteristics). Crucially, there was no difference in response rates of admits and nonadmits close to the admissions cutoff (*SI Appendix*, Fig. S1 and sections A.1 and A.2 give further information on the sample and measures).

In the United States, voting is public record and, as such, individual states make the list of registered voters in their state available to researchers. The voter file data that we use have been collected and collated by the data and analytics firm Data Trust. We use their snapshot of the nationwide voter file from 29 September 2017. Much like other large-scale voter-file vendors—like Catalist, L2, and Aristotle—Data Trust appends voting information from all 50 US states along with the District of Columbia into a single file. This appended dataset contains voting and registration histories of all registered voters in the United States—with ∼200 million voters contained therein. Whereas other firms share 1% samples of their voter files with researchers, we have the entire Data Trust file for all 200 million individuals, thus making it possible to match individuals who applied to participate in TFA. There is evidence that the Data Trust file is high quality; indeed, extant research (62–67) notes a high degree of fidelity between historical and contemporary measures found in Data Trust’s data and official reports of demographics, political partisanship, and turnout. This file also has good coverage on the inputs we use for matching and has relatively few potential duplicate individuals in the file. We use data on validated voter turnout from this file as our outcome of interest.

To merge the TFA applicants with their voting records in Data Trust, we used the fastLink package in R to probabilistically identify matches based on an applicant’s name, date of birth, and state of residence (68). Given that the survey we use began data collection in October 2015, we focus on turnout in the 2012 and 2014 elections (see *SI Appendix*, section A.5 for more details). We focus on the two most recent general elections to minimize the time elapsed between the elections in our voter file data and information on individuals’ current state of residence from the 2015 to 2016 survey. For all survey respondents, we searched for voting records in the current states of residence reported in the survey (more information about the matching process can be found in *SI Appendix*, section A.4). Following previous practice, if no voting record is found, we assume that person did not vote; this allows us to avoid issues with posttreatment bias (69–73).

Our core analyses are restricted to those who completed the survey conducted by Mo and Conn (33), as we have the most current information on geographic location for our population of interest among survey participants. However, we employ different geographic location data for estimating pretreatment effects and treatment effects. The majority of TFA applicants moved during the period between their application and the survey, and applicants who participated in TFA moved at even higher rates—perhaps because participating in TFA often involves moving to a different state. When we assess whether the state of residence information in the survey matches the state of residence indicated in the TFA’s administrative files from the application stage, we see that 61% of TFA participants moved states, compared to 50% of nonparticipants. Previous research has shown that increased mobility contributes to lower levels of voter turnout (74, 75). Moreover, the mobility of young people also makes record linkage tricky. Since our matching process blocks on state, inaccurate state information will lead to less accurate turnout estimates (76). We therefore rely on geographic information from TFA’s administrative file when estimating pretreatment effects (i.e., rate of political participation before any TFA national service could take place), as location information from the applications is an accurate source of an applicant’s location shortly before and when applying to TFA. When estimating treatment effects, we utilize the state information from the survey (33), as state of residence in 2015/2016 is more likely to be the state of residence during the 2012 and 2014 election for the cohorts for whom we can estimate posttreatment effects (2007 to 2012 application cycles) than the state of residence at the time of their application recorded in the admissions administrative file.

Participating in TFA is distinctly nonrandom. As individuals choose whether or not to participate in TFA, simply comparing the vote history of individuals who participate to those who do not—even if we control for observable characteristics—leaves open the possibility that any estimated effect of TFA would actually reflect selection bias rather than a systematic program effect. As such, to estimate the causal effect of TFA participation on voter turnout, we employ a natural experimental design. Specifically, we use a regression discontinuity design (RDD). RDDs are a natural experimental technique that have grown in popularity in recent years, especially in contexts where formal randomization is not/cannot be conducted. The design leverages scenarios where there is an arbitrary value of some variable—a cutoff—that sorts individuals into treatment and control groups; individuals on one side of the cutoff are in the treatment group, while individuals on the other are in the control group. As has been well established, regression discontinuity designs exploit continuity in potential outcomes around an arbitrary cutoff (35 to 40; see *SI Appendix*, section A.6 for more on the design’s application to our case). As long as the (often modest) assumption that the potential outcomes of the control and treatment groups are continuous around the cutoff is met, RDDs will provide causal estimates that benchmark remarkably well with randomized control trial estimates (41). As we show below, our RDD appears likely to satisfy the assumptions underlying this methodological approach.

The discontinuity that we leverage in this paper is found in the selection scores given to all of the young people who apply to participate in TFA. As background, in 2007 TFA instituted a selection process that assigns a continuous score to all applicants. This score represents TFA’s holistic assessment of how effective the applicant will be in the classroom based on the applicant’s application materials. This is based on applicants’ educational history, extracurricular activities, transcripts, personal statements, and interviews. Individuals who score above a certain preset score are recommended to be admitted to the TFA program, whereas those who score below the cutoff are not recommended to be admitted, although the score is not the only factor that determines whether an applicant will be admitted to the program (see *SI Appendix*, section A.3 for more details). Importantly, this cutoff and the weight given to the constituent parts that go into determining one’s score are not public knowledge to the interviewees and the interviewers. This provides us with a strong precondition for estimating the causal effect of TFA using an RDD: It is unlikely that individuals will be able to precisely sort around the admittance cutoff (39). As we would expect if this cutoff were (locally) sorting individuals as good as randomly, observable covariates (*SI Appendix*, Fig. S3) and the density of the running variable (*SI Appendix*, Fig. S4) are balanced at the cutoff. This approach allows us to overcome issues of selection bias, endogeneity, and/or simultaneous/reverse causation and estimate the causal effect of being chosen to participate in TFA (the ITT effect) and actually participating in TFA (the CACE). As is standard practice, the CACE is estimated via a fuzzy regression discontinuity design that instruments program participation with program selection (77).

## Supplementary Material

Supplementary File

## Data Availability

Nonproprietary anonymized replication data and code have been deposited in Harvard Dataverse (78). The nationwide voter file and TFA administrative data, which contain sensitive personally identifiable information, that are required to replicate our core findings can only be accessed through the Data Trust and TFA. The Data Trust data are proprietary and we have signed data use agreements that prohibit us from sharing them. The individual-level data can be provided by Bill Dune at The Data Trust pending scientific review and a completed material transfer agreement. Requests for the individual-level data should be submitted to bill.dunne@thedatatrust.com. The TFA admissions data are proprietary and we have signed data use agreements that prohibit us from sharing them. The individual-level data can be provided by TFA pending scientific review and a completed material transfer agreement. Requests for the individual-level data should be submitted to research@teachforamerica.org. All other data needed to evaluate the conclusions in this paper are present in this paper and/or supporting information.

## Footnotes

^*^This was TFA’s mission statement at the start of the organization; however, the language of their core mission has recently changed. With that said, the noted mission or goals in several of TFA’s regional office uses this original language. For example, see TFA San Diego’s website (https://www.teachforamerica.org/where-we-work/san-diego/our-work [accessed 15 June 2022] and TFA Eastern North Carolina’s website (https://www.teachforamerica.org/where-we-work/eastern-north-carolina/our-work [accessed 15 June 2022]).

^†^When examining the 2007 to 2015 cohorts, we find that 87% of those who matriculated into the program completed the full 2-y program commitment.
