# Supplementary material for: Civilian national service programs can powerfully increase youth voter turnout
Source: Proc Natl Acad Sci U S A. 2022 Jul 11;119(29):e2122996119. doi: 10.1073/pnas.2122996119 (PMC9304004; doi:10.1073/pnas.2122996119)
Supplement: Supplementary File [file pnas.2122996119.sapp.pdf]

Supplemental Information

Civilian National Service Programs Can Powerfully Increase Youth Voter Turnout

Contents

A Additional Information on the Data and Methods 3
A.1 Data Details 3
A.2 Sample Characteristics 6
A.3 Identifying Assumptions 7
A.4 Matching Procedure 10
A.5 Outcome Measures 13
A.6 Regression Discontinuity Analysis 14
A.7 Alternative Presentation of Main Results 16
A.8 Tests of Treatment Effect Heterogeneity 18
A.9 Alternative Dependent and Independent Variables 19
A.10 Proposed Congressional Legislation on National Service 27

B Supplementary Text 32
B.1 The Many Mechanisms by Which TFA May Increase Voter Turnout 32
B.2 Careers of Non-Admits 34

List of Figures

S.1 Response Rates 7
S.2 First Stage Results 8
S.3 Reduced Form Estimates of Pre-Treatment Characteristics 9
S.4 Admission Score Distribution 9
S.5 Graduation Year to Proxy Birth Year 11
S.6 Effect of TFA Experience on Voter Turnout 17
S.7 Tests of Treatment Effect Heterogeneity 18
S.8 Effect of TFA Experience on Voter Registration 19
S.9 Effect of TFA Experience on Voter Turnout (Share of Elections) 20
S.10 Effect of Minimal TFA Experience on Voter Turnout 22
S.11 Effect of 1-Year TFA Experience on Voter Turnout 23
S.12 Effect of TFA Experience on Pre-Treatment Turnout (2008-2014 Elections) 24
S.13 Effect of TFA Experience on Post-Treatment Turnout (2008-2014 Elections) 25
S.14 Effect of TFA Experience on Turnout (Completed Program as Treatment) 26
S.15 Sector of First Job Held Since 2007 of Non-Participants 35
S.16 Sector of Second Job Held Since 2007 of Non-Participants 36
S.17 Sector of Third Job Held Since 2007 of Non-Participants 37

List of Tables

S.1 Demographic Characteristics of Sample 6
S.2 Recent Legislation on National Service 28
S.3 Comparing Teachers and Non-Teachers 34

A Additional Information on the Data and Methods

A.1 Data Details

The original file of all applicants who advanced to the final stage of the TFA admissions process contained 134,808 observations. 5,463 applicants with contact restrictions, 7,221 applicants with invalid email addresses, 1,568 duplicate cases (linked to applicants who applied to TFA multiple times), and 88 applicants with no selection score were removed. Regarding the survey of TFA applicants, to ensure applicants who applied more than once would only be contacted once, Mo and Conn (2018) preserved contact information for only the most recent application year (*1*). The remaining 139 applicants were removed when checking for duplicate errors. They utilized the application file only, and did not update the contact information for alumni to ensure that the share of contact information errors in the file would be the same for admits and non-admits.

All of these applicants were also targeted by Mo and Conn (2018) between October 1, 2015 and March 31, 2016 to complete an online survey; 32,595 of these targeted applicants (27.1 percent) responded to some portion of this survey and 24,886 applicants (21.6 percent) completed the survey (*1*). After removing non-citizens and individuals who did not provide a current state of residence, we were left with a sample of 28,662 potential voters.

Details of the data we received from Teach For America, as well as the items we used responses (indicated with the label "Survey item") from the original online survey administered between October 1, 2015 and March 31, 2016, are provided below. Exact question wording and information on our response recoding of question items that were recoded are provided.

Application Information

1. *Application Year* - The cohort an applicant was applying for was provided. (Response Options: 2007, 2008, 2009, 2010, 2011, 2012, 2013, 2014, and 2015)

2. *Graduation Year* - The year in which the applicant graduated with a bachelor's degree. (Open-ended response (yyyy))

3. *Admission Score* - Applicant's final admission score was provided. Only individuals who made it to the final round of the admission process received an admission score, and our target sample focused on individuals that made it to this final round only.

4. *Admission Cutoff Score* - Information on the cutoff score was provided for each application year. To combine cohorts, we standardized each year such that the cutoff is at 0, higher values indicate scoring better, and values can be interpreted as the number of standard deviations away from the cutoff the applicant was.

5. *Admission Decision* - Information on whether an applicant was admitted into TFA was provided. (Response Options: 0 = No; 1 = Yes)

6. *Matriculation Decision* - Information on whether an admitted applicant matriculated into TFA was provided. (Response Options: 0 = No; 1 = Yes)

7. *Email Information* - Up to two email addresses were provided for each applicant.

8. *Current Address* - Mailing address shared by each applicant when applying to TFA was provided. We used the state information from the mailing address.

9. *University* - Undergraduate university or college each applicant graduated from was provided. We used the state location of the university or college.

10. *Mobile Phone* - Mobile phone number shared by each applicant when applying to TFA was provided. We used the area code to identify state information.

11. *State* - Survey item: "In which state do you currently reside?" [Dropdown menu with all 50 states, Washington, D.C., and US territories]

Demographic Characteristics

1. *Age* - The applicant data provided by TFA contained information on applicant birth date, which could be used to compute an applicant's age at the time of the survey. The survey also asked: "What year were you born?" Respondents indicated the year in which they were born, and this was recoded such that the variable indicates their age in years. For all analyses aside from descriptive analyses, the variable was coded to be between 0 and 1.

2. *Female* - The applicant data provided by TFA contained information on applicant gender. The survey also asked: "What is your gender?" (Response Options: 0 = Male; 1 = Female)

3. *Ethnicity* - The applicant data provided by TFA contained information on applicant race/ethnicity. The survey also asked: "What racial or ethnic group best describes you?" (Response Options: 1 = White; 2 = Black or African American, 3 = Hispanic or Latino; 4 = Native American; 5 = Asian; 6 = Native Hawaiian or Pacific Islander; 7 = Other (please specify:))

   (a) *White* (Response Re-Coding: 0 = All Else; 1 = White)

   (b) *Black* (Response Re-Coding: 0 = All Else; 1 = Black or African American)

   (c) *Hispanic* (Response Re-Coding: 0 = All Else; 1 = Hispanic or Latino)

   (d) *Asian* (Response-Coding: 0 = All Else; 1 = Asian)

4. *Received Pell Grant* - The applicant data provided by TFA contained information on whether the applicant qualified to receive a Pell Grant (e.g., financial aid) in college. (Response Options: 0 = No; 1 = Yes)

5. *Social Class* - Survey item: “When you were growing up, would you describe your family as belonging to the...?” (Response Options: 1 = Upper Class; 2 = Upper Middle Class; 3 = Lower Middle Class; 4 = Upper Lower Class; 5 = Lower Class)

   (a) *Upper Class* - “Upper Class” (Response Re-Coding: 0 = All Else; 1 = Upper Class)

   (b) *Upper Middle Class*(Response Re-Coding: 0 = All Else; 1 = Upper Middle Class)

   (c) *Lower Middle Class* (Response Re-Coding: 0 = All Else; 1 = Lower Middle Class)

   (d) *Upper Lower Class* (Response Re-Coding: 0 = All Else; 1 = Upper Lower Class)

   (e) *Lower Class* (Response Re-Coding: 0 = All Else; 1 = Lower Class)

6. *Religiosity* - Survey item: “What is your religious affiliation?” (Response Options: 1 = Roman Catholic; 2 = Protestant; 3 = Orthodox (Russian/Greek/etc.); 4 = Jewish; 5 = Muslim; 6 = Hindu; 7 = Buddhist; 8 = Agnostic; 9 = Atheist; 10 = Not Religious; 11 = Some Other Religion (please specify:))(Response Re-Coding: 0 = Agnostic, Atheist, or Not Religious; 1 = Any Denomination Selected or Given)

A.2 Sample Characteristics

Table S.1 shows descriptive statistics on the sample's demographic characteristics. The source of each variable included in the summary table is indicated in parentheses. For variables from the survey, each statistic represents the roughly 30,000 people who took the survey. For variables from the application (app), each statistic represents the roughly 120,000 people for whom we obtained application data.

Table S.1: Demographic Characteristics of Sample

Variable	Mean	SD	Min	Max
**Survey Age Variables**				
Birth Year (survey)	1987.10	5.72	1950	1999
Age at Application (survey)	24.43	5.64	12	65
Less than 25 at application (survey)	0.78	0.41	0	1
**App Age Variables**				
Birth Year (app)	1987.24	5.72	1940	1998
Age at Application (app)	24.32	5.57	13	71
Less than 25 at application (app)	0.79	0.41	0	1
**Census Region (survey)**				
West	0.22	0.42	0	1
South	0.36	0.48	0	1
Midwest	0.18	0.38	0	1
Northeast	0.24	0.43	0	1
**Other Demographic Variables**				
Received Pell Grant (app)	0.34	0.47	0	1
Female (app)	0.72	0.45	0	1
White (app)	0.64	0.48	0	1

A.3 Identifying Assumptions

First, we check for differential attrition by examining the distribution of response rates at the threshold for admission. As we show in Figure S.1, response rates are continuous at the admission score cutoff, allaying concerns that our estimates could be biased by differences in the probabilities "treated" and "comparison" applicants responded to our survey. Our reduced form estimate of the effect of scoring above the TFA cutoff on survey completion rates is 0.0002 and statistically indistinguishable from zero ($p = 0.978$). We conclude that applicants near the margin for admission were equally likely to take our survey.

Fig. S.1: Response Rates

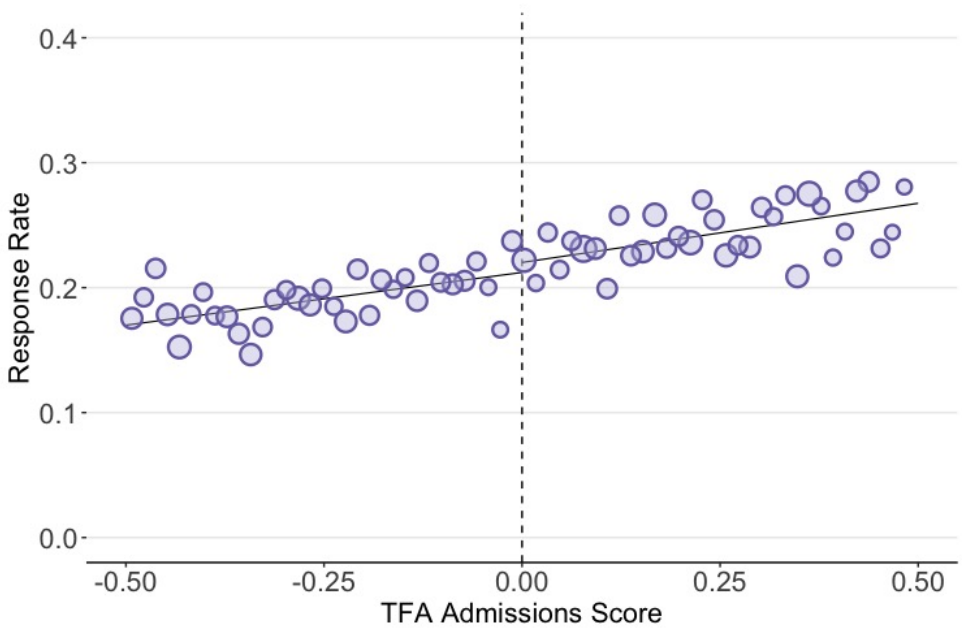

Notes: This figure plots survey response rates by admission score and includes a 95 percent confidence interval. The dots that represent the binned averages are sized to indicate number of observations. We re-centered the admission score distribution such that zero represents the cutoff score for each year. We then standardized admission scores by year. The bin size is 0.05.

Secondly, we show in Figure S.2, the probability an individual participates in TFA is a discontinuous function of that selection score. Specifically, among survey respondents, individuals who score just above TFA's admission cutoff, c, are 21.99 percentage points more likely to become TFA teachers than those who just barely miss the threshold for admission ($F = 64.41$). As such, the TFA cutoff acts as a strong instrument to isolate plausibly random variation in an individual's TFA participation status.

Thirdly, we test for differences in the observable characteristics of individuals who scored just on either side of the admission score cutoff to ensure that individuals just below the cut-

Fig. S.2: First Stage Results

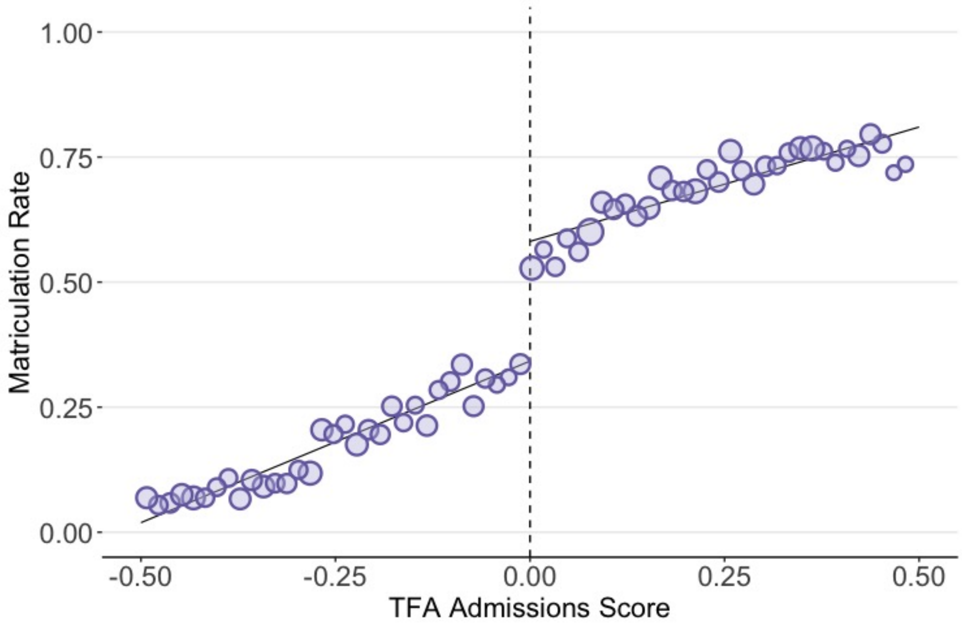

Notes: Figure 1 plots the fraction of individuals who matriculated in TFA by admission score. We re-centered the admission score distribution such that zero represents the cutoff score for each year. We then standardized admission scores by year. In each figure, the bin size is 0.015. The circle size represents the density of observations within each bin. The sample includes all TFA applicants who responded to our survey.

off are similar to those just above the cutoff. As we summarize in Figure S.3, the reduced form estimate of the jump in each baseline applicant characteristic at the cutoff among survey respondents is zero, signifying that there are no differences in observable applicant characteristics at the cutoff. (We likewise find that observable pre-treatment measures trend smoothly at the cutoff when we use the full pool of applicants to TFA instead of just those who responded to our survey.)

Finally, we test for possible manipulation of selection scores both visually and empirically (Figure S.4). The TFA admission officers do not share the admissions score cutoff with interviewers. Consistent with this policy, neither of these exercises suggests either selectors or applicants were aware of the cutoff. Specifically, when we conduct a test for score manipulation (*2*), we observe that the density of admission scores is continuous at the cutoff, both for the entire TFA applicant pool ($p = 0.754$) and for the individuals who responded to the survey ($p = 0.925$).

Fig. S.3: Reduced Form Estimates of Pre-Treatment Characteristics

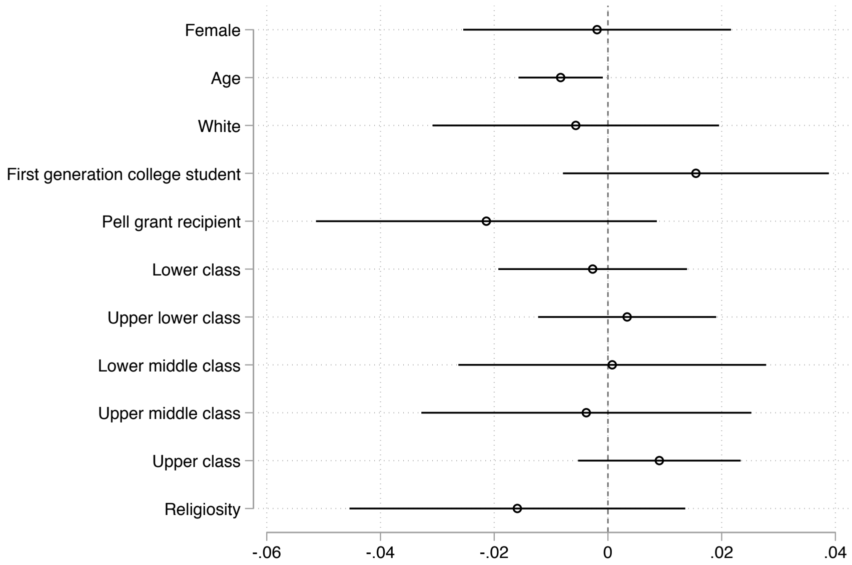

Notes: This figure displays reduced form coefficients estimated with 95 percent confidence intervals surrounding point estimates. The sample includes all TFA applicants who responded to this survey.

Fig. S.4: Admission Score Distribution

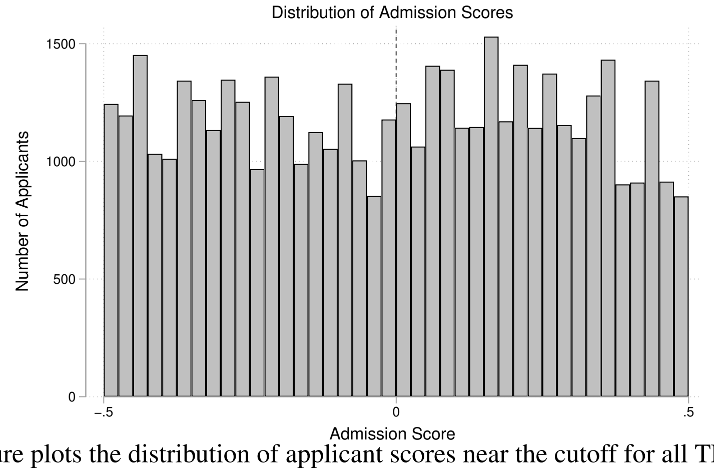

Notes: This figure plots the distribution of applicant scores near the cutoff for all TFA applicants to the 2007-2015 cohorts. We likewise observe that the density of admission scores is continuous at the cutoff when we restrict the sample to the individuals who responded to the survey.

A.4 Matching Procedure

To match the TFA applicants with their records in the voter file, we used the fastLink algorithm (see (*3*) for details on the algorithm and its application to linking with voting records). We relied on three pieces of information about applicants to locate their vote histories: their name, their birth year, and their state of residence. The fastLink algorithm calculates the posterior likelihood of a link between an applicant and an entry in the voter file. We matched fully on first name and sex (i.e. the sex and first name of a voter file entry and applicant must be exactly the same to count towards the match likelihood) and partially on last name and birth year (i.e. voter file entries and applicants are considered better or worse matches based on the string/year distance between their last names and birth years). Matches with a posterior probability of less than 0.85 were discarded.[1]

First name, last name, and sex were available for nearly all applicants from TFA records. Birth year was available from TFA and from the Mo and Conn (2018) survey (*1*). Data on birth year from the survey is available at similar rates for admitted and non-admitted applicants, but data on birth year from TFA is missing for many non-admitted applicants in the four application cohorts between 2010 and 2013. For this reason, many results presented here rely on matches based on birth year information from the survey.

In the analyses which use the TFA birth year from the administrative data, we drop the birth year information for the 2010-2013 cohorts and proxy for respondents' birth years by subtracting 22 years from their date of college graduation, as birth year information is only provided for TFA participants in those years. With graduation year missing for only 3 applicants and a plurality of applicants graduating from college at age 22, this proxy method allows us to approximate birth year for all applicants in a uniform manner. Importantly, this proxy method produces an estimate of birth year that is similarly accurate on both sides of the cutpoint, as illustrated in Figure S.5, which shows the proportion of applicants with birth years equal to, one year from, or 2-3 years from the proxy measure of age on either side of the cutpoint. There is no statistically significant difference at the cutpoint in the accuracy of the proxy measure in any of these cases ($p = .39, .97, .99$ respectively).

We carried out the matching process separately for each state's voter file, only looking for matches between each state's voter registrants and applicants we identified as potential residents of that state. Potential states of residence were identified based on four possible sources: the state of residence reported on the Mo and Conn (2018) survey (*1*), the state of residence reported on the TFA application, the area code of the cell phone number on the TFA application, and the state of the applicant's university. We searched for matches for an applicant in every state linked to them by at least one of these four sources.

Analyses reported in the paper use one of two sets of matches: those from the state each applicant reported in the survey, and those from a combination of application-based sources (which we call the "hybrid" strategy). For the hybrid strategy, we first use matches from the

[1] The threshold of 0.85 is the value chosen by the fastLink package authors as the function's default, and it is the threshold preferred by the authors in a publication in a similar application to the context here (*4*).

Fig. S.5: Graduation Year to Proxy Birth Year

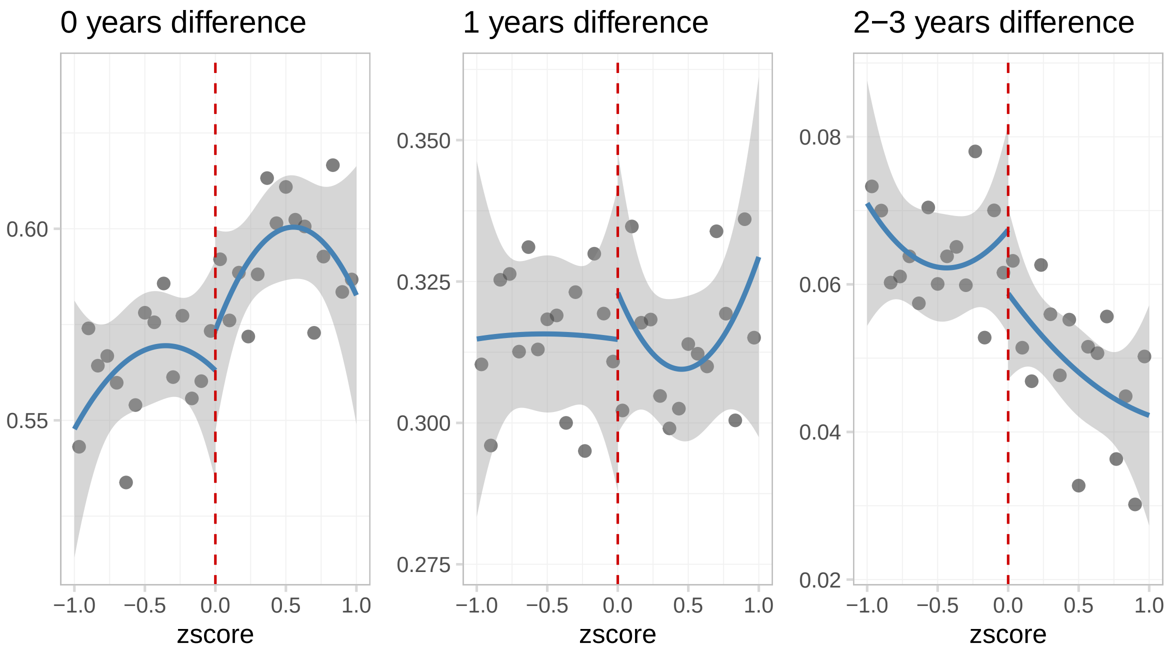

Notes: Each panel shows the proportion of applicants at a given application score with birth years (reported in the survey) which are 0 years, 1 year, or 2-3 years different from the proxy measure, calculated by subtracting 22 years from the applicant's year of graduation.

state listed as an applicant's current address in their application; if there is no match available from the state in their current address, we use matches from the state of their university, then matches from the state of their cell phone number's area code.

The Data Trust voter file for any given state includes records for people who have registered to vote in that state and have not been purged from the voter file, as well as some records from commercial sources. The file therefore includes a mix of registered voters and people who are not or are no longer registered to vote. In all, 70.5% of survey respondents were matched to a record in the voter file in the state of residence they indicated on the survey. For the full sample of 120,329 final-round applicants, we had up to three different sources of location data in the TFA administrative data: their addresses, phone numbers, and universities. After restricting the sample to voting-eligible applicants, we found potential states of residence in the TFA administrative data for more than 99% of applicants through these sources. For the 59% of applicants who were linked to multiple states through these sources, we searched for records in each state; if multiple records were discovered, we prioritized them in the following order: current address, university location, and cell phone area code. We found matches for 41% of

voting-eligible applicants in the states of residence indicated by their application. The match rate is lower because the application state information is more likely to be outdated at the time of the voter file data compilation in 2017.

A.5 Outcome Measures

The key outcome variable used here measures whether an applicant voted in either the 2012 or 2014 national general election. Though data on earlier elections is available, the removal of voters due to state maintenance of voter files makes it more difficult to match applicants in earlier years. Applicants who were ineligible to vote in both elections due to their age are coded as missing.

To calculate post-treatment effects, we use an outcome measure capturing whether an applicant would have completed 2 years in TFA at the time of each election if admitted. For example, someone who applied in 2010 and was not accepted would have completed the program in 2013 if admitted. They would not have been "post-TFA" for the 2012 election, so this election is not considered in the outcome measure. However, they would have been "post-TFA" in the 2014 election. The outcome measure would therefore be coded as 1 if the applicant voted in 2014 and 0 if they had not. Applicants who would not have completed TFA by the 2014 election are coded as missing.

To calculate pre-treatment effects, the outcome measure captures whether an election took place before the applicant applied to TFA. Any elections that took place before the application are not considered.

A.6 Regression Discontinuity Analysis

We employed a fuzzy regression discontinuity design (RDD) to estimate the causal effect of TFA participation on voter turnout. Each applicant i who advanced to the final round of the TFA admission process received a selection score, X_i. We define our instrument, Z_i, as follows:

$$Z_i = \begin{cases} 1, & \text{if } X_i \geq c \\ 0, & \text{if } X_i < c. \end{cases} \tag{1}$$

The results referred to as the "ITT," or intent to treat, capture the causal effect of having an application score above the cutpoint on turnout. The results referred to as the "CACE," or complier average causal effect, capture the causal effect of participation in TFA on compliers, instrumented by an application score above the cutpoint. Compliers are applicants who would matriculate if and only if they receive a score above the cutpoint; because the application score is not a perfect predictor of matriculation on either side of the cutpoint, we employ a "fuzzy" regression discontinuity. The estimates are local to applicants with scores at the cutpoint.

We estimate results using the "rdrobust" package in R (*5*). The rdrobust package selects a bandwidth for analysis to optimize mean squared error, calculates the CACE or ITT using a local polynomial estimator, adjusts the calculated CACE/ITT for bias induced by bandwidth selection, and calculates robust standard errors that account for the bias correction. We use a triangular kernel to weight observations; results are substantively similar with other weighting schemes.

To estimate the effect of having a score just above the cutpoint compared to one just below, we estimate an equation of the following form:

$$ITT = \lim_{Z \to z^+} E[Y|Z = z] - \lim_{Z \to z^-} E[Y|Z = z]$$

where $E[Y|Z = z]$ represents the conditional expectation of turnout at a particular application score. $\lim_{Z \to z^+} E[Y|Z = z]$, then, represents the limit of the conditional expectation function as the application score approaches the cutpoint from above; the second half of the equation is the limit as it approaches the cutpoint from below. The ITT, then, could be interpreted as the difference in expected turnout between a just-admitted and a just-rejected applicant with scores exactly at the cutpoint.

In a fuzzy regression discontinuity setup as we employ here, the complier average causal effect (CACE) can be represented like this:

$$CACE = \frac{\lim_{Z \to z^+} E[Y|Z = z] - \lim_{Z \to z^-} E[Y|Z = z]}{\lim_{Z \to z^+} E[M|Z = z] - \lim_{Z \to z^-} E[M|Z = z]}$$

where M is a binary variable measuring whether an applicant matriculated in Teach for America. That is, we divide the ATT by the difference in the expected probability of matriculating in TFA for applicants with scores just above and just below the cutpoint.

We use the following code to estimate the ITT: `rdrobust(y=dv, x=z, all=TRUE)`, where "dv" is the outcome variable and "z" is the application score. We use the following code to estimate the CACE: `rdrobust(y=dv, x=z, fuzzy=matric, all=TRUE)`, where "matric" is an indicator for whether an applicant matriculated in TFA.

A.7 Alternative Presentation of Main Results

Figure S.6 below presents the key results in the main text, showing turnout levels across levels of the running variable rather than summarizing the effects in a coefficient plot.

Fig. S.6: Effect of TFA Experience on Voter Turnout

(a) Pre-Treatment, Match 1

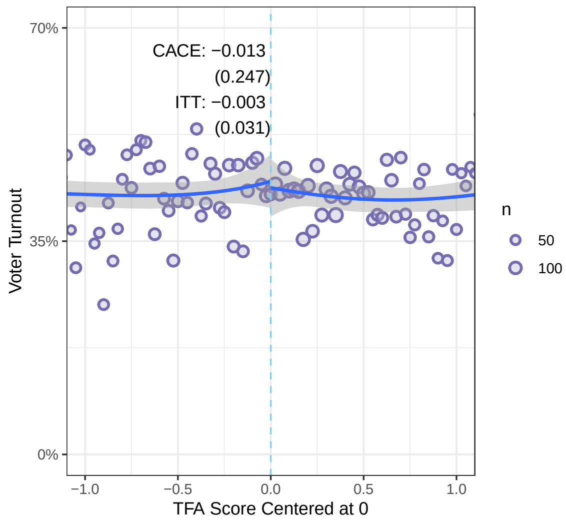

(b) Pre-Treatment, Match 2

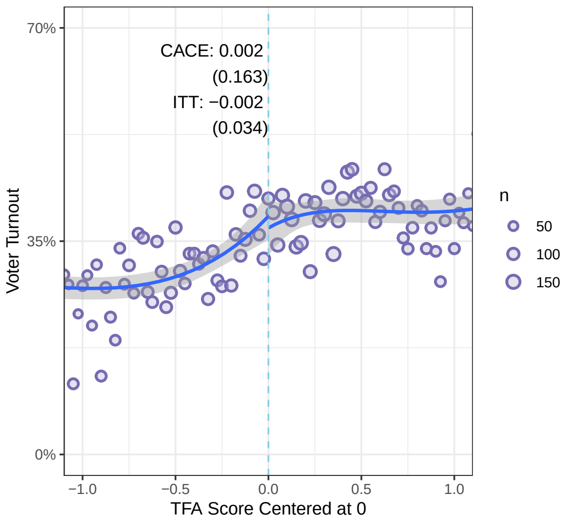

(c) Post-Treatment, Match 1

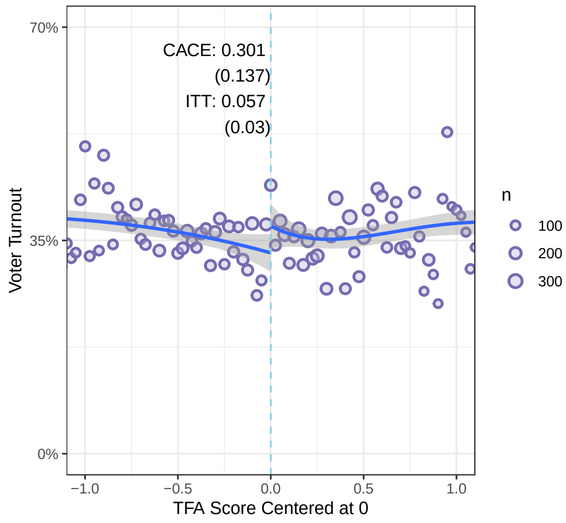

(d) Post-Treatment, Match 2

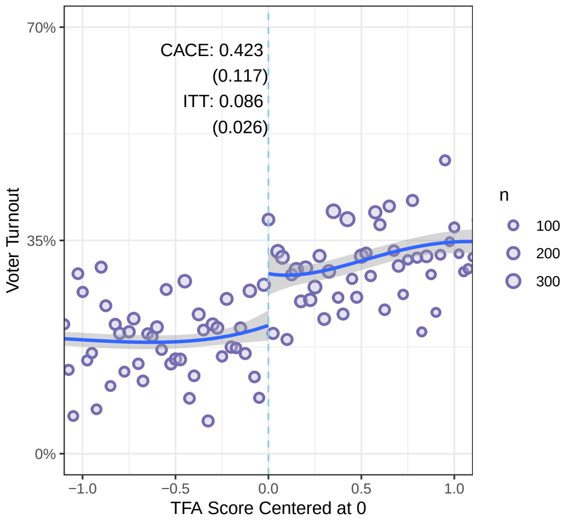

Note: Points to the right of 0 are those who were above the admittance threshold, whereas those to the left are those that are below it. Each sub-figure reports the complier average causal effect (i.e. the CACE) and the intention to treat effect (i.e. the ITT) annotated above the TFA admittance threshold. Therein, the standard errors are in parentheses below the coefficient estimates. The figures show the average levels of voter turnout by selection score. Lines are 4th degree polynomials fitted separately on either side of the cutpoint (*6*). Binned values are sized to indicate number of observations.

A.8 Tests of Treatment Effect Heterogeneity

Figure S.7 tests for treatment effect heterogeneity by race, gender, region of origin, and federal Pell Grant status (a proxy of socioeconomic status). When it comes to treatment effect heterogeneity, these effects are similar for whites and non-whites and across several geographic areas in the United States, but appear to be larger for males than females and perhaps for non-Pell Grant recipients than Pell Grant recipients.

Fig. S.7: Tests of Treatment Effect Heterogeneity

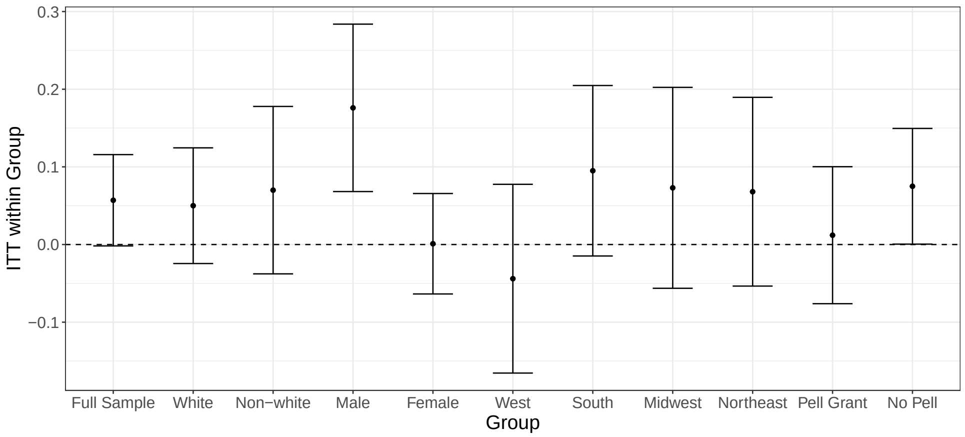

Notes: Coefficient plots for the ITT across the individual characteristics listed on the x-axis. Points show effects and bars show 95% CIs.

A.9 Alternative Dependent and Independent Variables

This section first replicates the analyses in the main text with alternative dependent variables. First, Figure S.8 shows the effect of TFA acceptance and matriculation on registration to vote. This variable measures whether an applicant was located in the voter file as registered to vote at any point covered by our data. The analysis using matching strategy 2 suggests a significant positive effect on registration; the effects found using strategy 1 are not significant, though they point in the same direction.

Fig. S.8: Effect of TFA Experience on Voter Registration

(a) Match 1 (b) Match 2

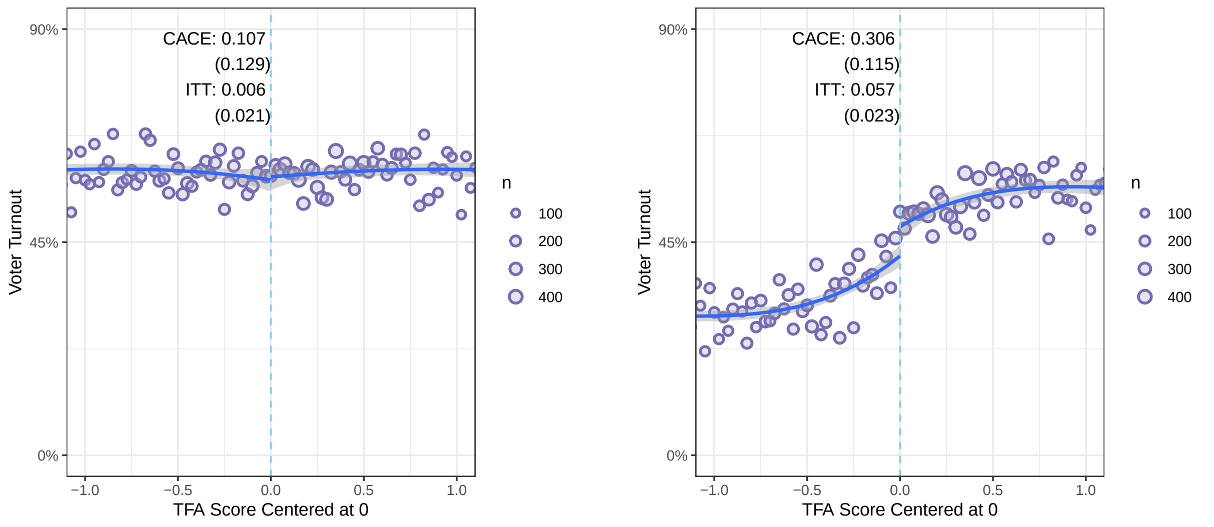

Note: Points to the right of 0 are those who were above the admittance threshold, whereas those to the left are those that are below it. Each sub-figure reports the complier average causal effect (i.e. the CACE) and the intention to treat effect (i.e. the ITT) annotated above the TFA admittance threshold. Therein, the standard errors are in parentheses below the coefficient estimates. The figures show the average levels of voter registration by selection score. Lines are 4th degree polynomials fitted separately on either side of the cutpoint (*6*). Binned values are sized to indicate number of observations.

Figure S.9 shows the effect of TFA acceptance and matriculation on the proportion of elections in which an applicant voted, after they (would have) completed TFA. Both models suggest significant positive effects: applicants narrowly accepted to TFA voted in appreciably more elections than their peers who were narrowly rejected.

Next, we turn to the effects of TFA on turnout before the program is complete. The analyses in the main text focuses on effects of completing the program (i.e., completed their two years of service). Figure S.10 shows the effects of TFA on turnout for those who are only a few months into the program: 2012 applicants in the 2012 election, and 2014 applicants in the 2014

Fig. S.9: Effect of TFA Experience on Voter Turnout (Share of Elections)

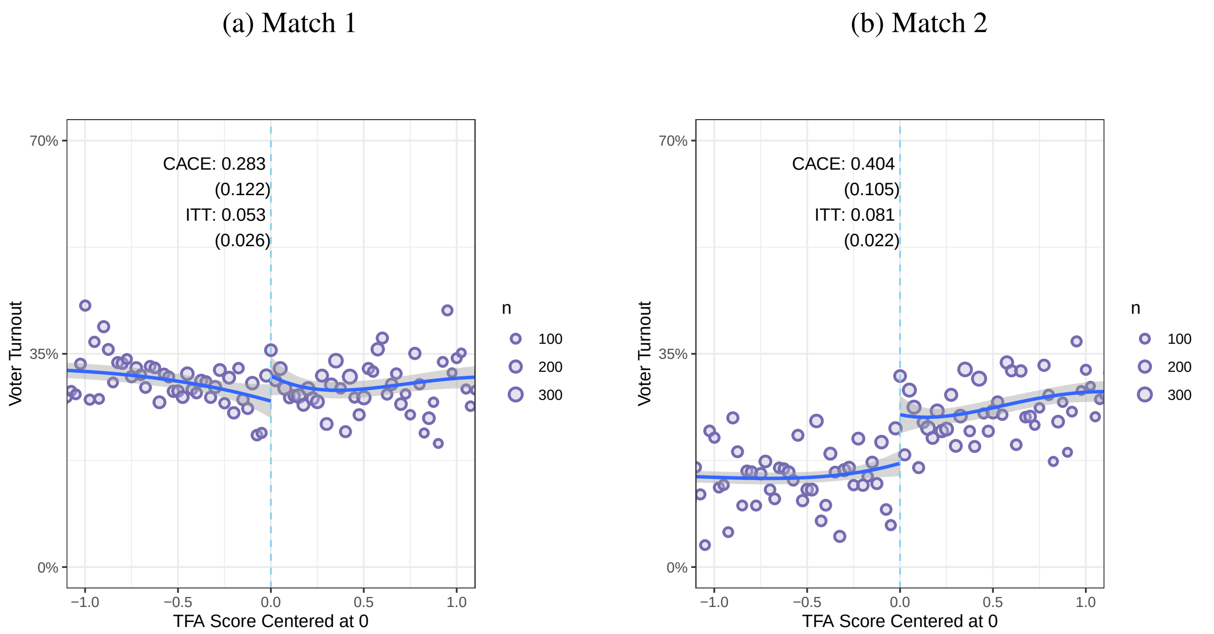

Note: Points to the right of 0 are those who were above the admittance threshold, whereas those to the left are those that are below it. Each sub-figure reports the complier average causal effect (i.e. the CACE) and the intention to treat effect (i.e. the ITT) annotated above the TFA admittance threshold. Therein, the standard errors are in parentheses below the coefficient estimates. The figures show the average levels of voter turnout by selection score. Lines are 4th degree polynomials fitted separately on either side of the cutpoint (*6*). Binned values are sized to indicate number of observations.

election. For one model, the 2012 cohort using match strategy 2, there is a positive effect of TFA selection/participation on turnout; for the other models, however, there is no evidence of a positive effect. Overall, the program does not seem to have consistent positive effects on voter turnout after only a few months of participation.

Figure S.11 shows the effect of having spent a full year in the program: 2011 applicants in the 2012 election and 2013 applicants in the 2014 election. Again, there does not seem to be evidence of a positive effect of treatment on participation before receiving the full "dosage" of two years' participation. These results should be interpreted with caution. The samples, limited to a single cohort each, are substantially smaller than those in the analyses reported in the main text. They are also more prone to measurement error because they rely on only 1 election to measure turnout, rather than the longer periods used for the dependent variables in the main text. However, in sum, we are not able to detect consistent positive effects of TFA on voter turnout when participants are still in the early stages of the program.

We next expand the scope of elections included in the dependent variable. Analyses in the main text are restricted to the 2012 and 2014 elections, as regular voter file maintenance means many records of people voting in earlier elections have been purged from our data. Figure S.14

shows results including the 2008 and 2010 elections. The post-TFA results are nearly identical to the results in the main text, which reflect the fact that no applicants were "post-TFA" in the 2008 election, as well as that few people in our sample voted in 2010 but not later. The pretreatment results show different point estimates for the ITT and CACE than the results in the main text, but as in the main text, there is no significant pretreatment effect of a barely-passing score on turnout for three of the four specifications.

Finally, we repeat analyses replacing the "matriculation" variable compliance indicator with an indicator for whether a participant completed the TFA program. This data is only available for those in the application cohorts 2007-2011. This analysis addresses the concern that the treatment effects of the matriculation variable are being driven by matriculated participants who left the program in the course of completion. However, the effect is stronger using the completion indicator than the matriculation results in the main text, a result consistent with the notion that participants who complete the program are driving the main findings.

Fig. S.10: Effect of Minimal TFA Experience on Voter Turnout

(a) 2012 Cohort, Match 1

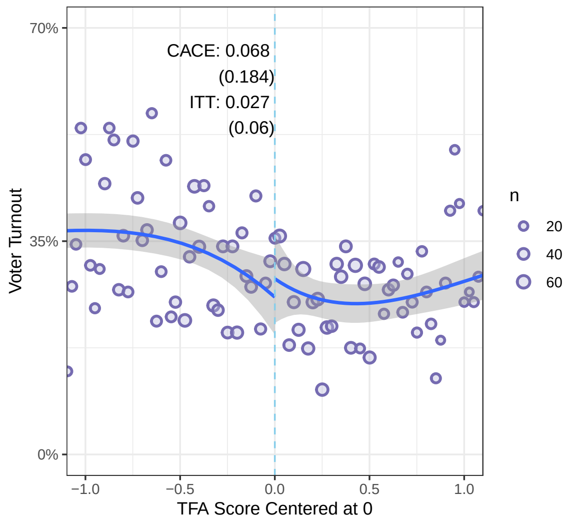

(b) 2012 Cohort, Match 2

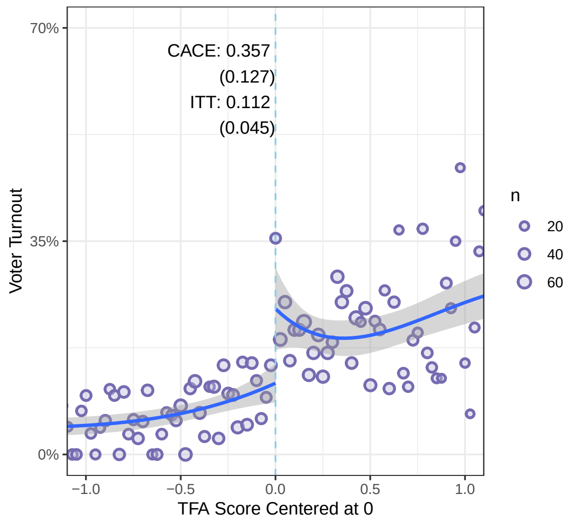

(c) 2014 Cohort, Match 1

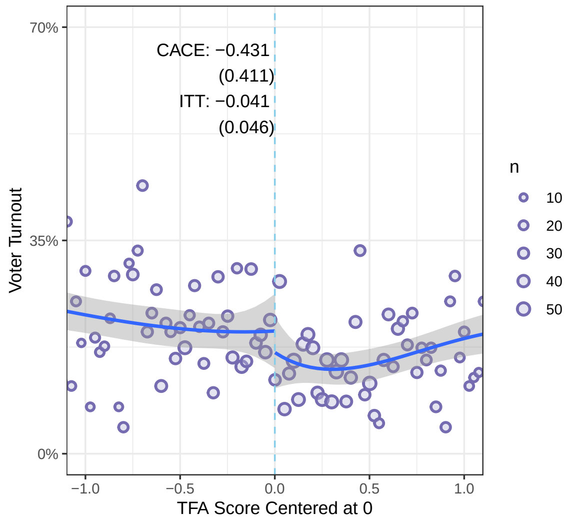

(d) 2014 Cohort, Match 2

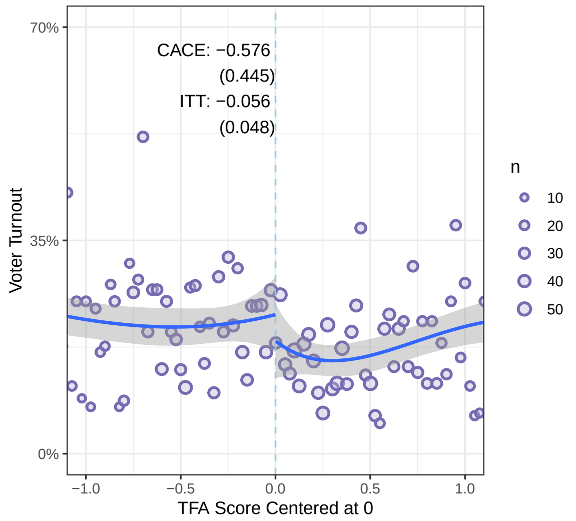

Note: Points to the right of 0 are those who were above the admittance threshold, whereas those to the left are those that are below it. Each sub-figure reports the complier average causal effect (i.e. the CACE) and the intention to treat effect (i.e. the ITT) annotated above the TFA admittance threshold. Therein, the standard errors are in parentheses below the coefficient estimates. The figures show the average levels of voter turnout by selection score. Lines are 4th degree polynomials fitted separately on either side of the cutpoint (*6*). Binned values are sized to indicate number of observations.

Fig. S.11: Effect of 1-Year TFA Experience on Voter Turnout

(a) 2011 Cohort, Match 1

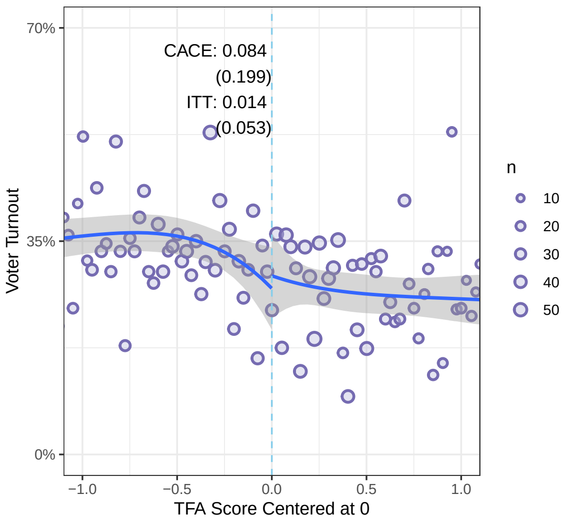

(b) 2011 Cohort, Match 2

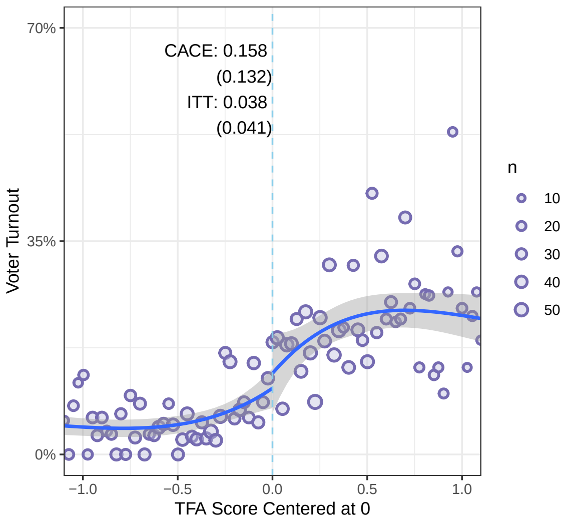

(c) 2013 Cohort, Match 1

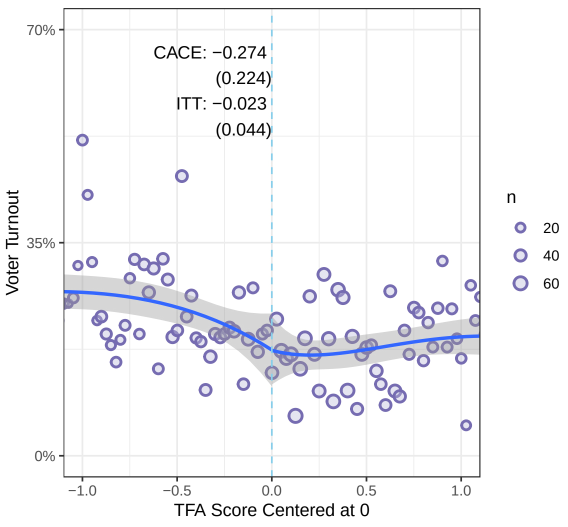

(d) 2013 Cohort, Match 2

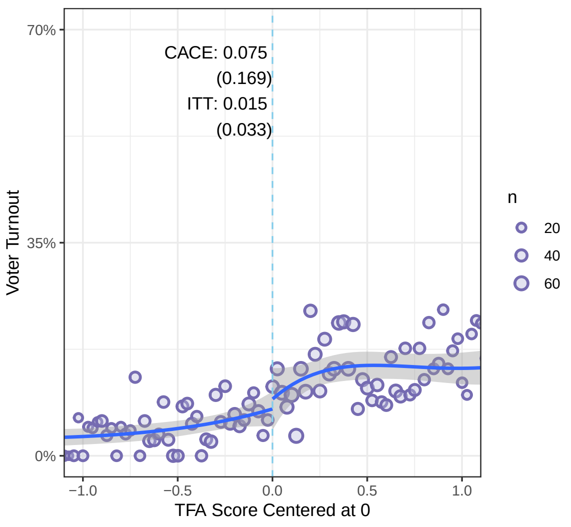

Note: Points to the right of 0 are those who were above the admittance threshold, whereas those to the left are those that are below it. Each sub-figure reports the complier average causal effect (i.e. the CACE) and the intention to treat effect (i.e. the ITT) annotated above the TFA admittance threshold. Therein, the standard errors are in parentheses below the coefficient estimates. The figures show the average levels of voter turnout by selection score. Lines are 4th degree polynomials fitted separately on either side of the cutpoint (*6*). Binned values are sized to indicate number of observations.

Fig. S.12: Effect of TFA Experience on Pre-Treatment Turnout (2008-2014 Elections)

(a) Pre-Application, Match 1 (2008-2014)

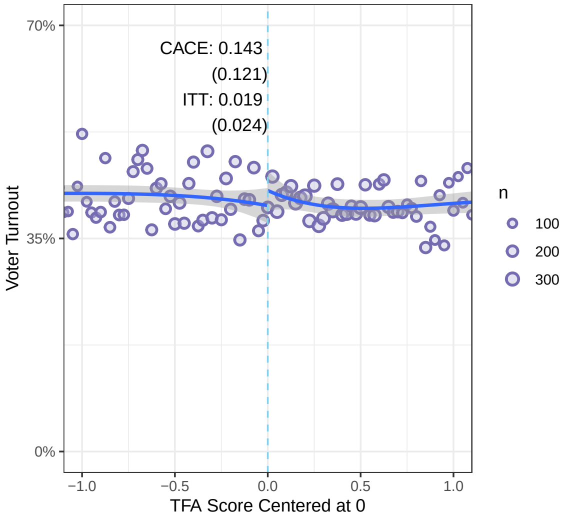

(b) Pre-Application, Match 2 (2008-2014)

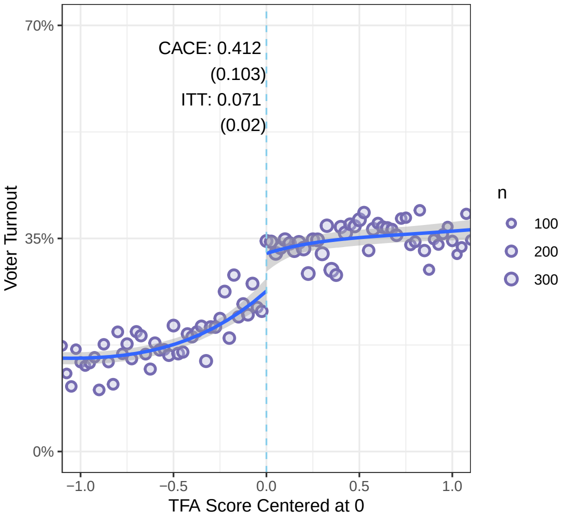

(c) Pre-Application, Match 1 (2010-2014)

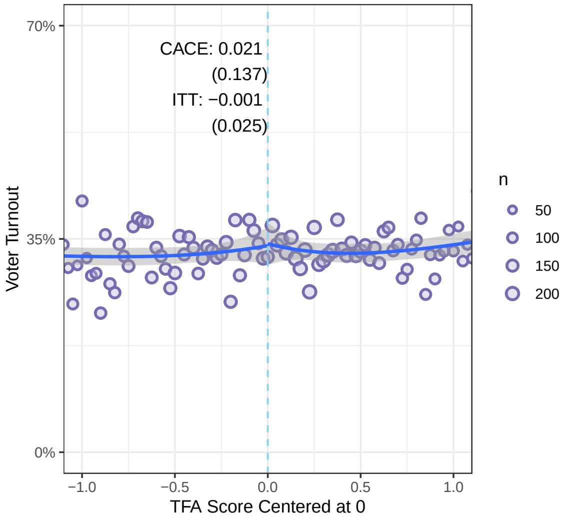

(d) Pre-Application, Match 2 (2010-2014)

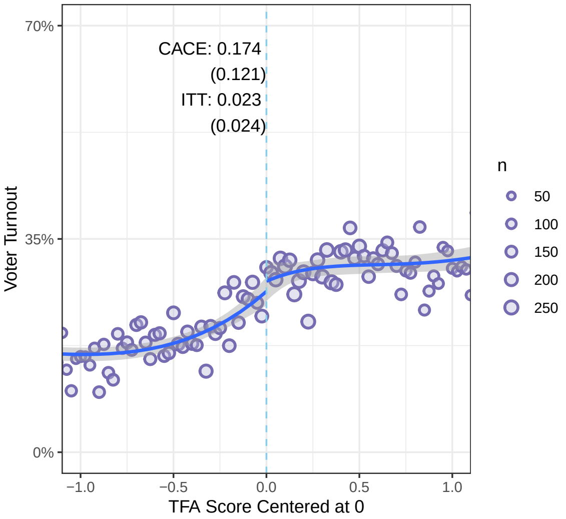

Note: Points to the right of 0 are those who were above the admittance threshold, whereas those to the left are those that are below it. Each sub-figure reports the complier average causal effect (i.e. the CACE) and the intention to treat effect (i.e. the ITT) annotated above the TFA admittance threshold. Therein, the standard errors are in parentheses below the coefficient estimates. The figures show the average levels of voter turnout by selection score. Lines are 4th degree polynomials fitted separately on either side of the cutpoint (*6*). Binned values are sized to indicate number of observations.

Fig. S.13: Effect of TFA Experience on Post-Treatment Turnout (2008-2014 Elections)

(a) Post-TFA, Match 1 (2008-2014)

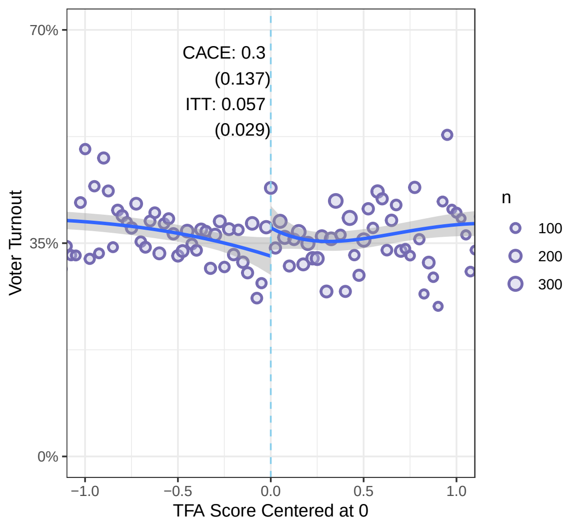

(b) Post-TFA, Match 2 (2008-2014)

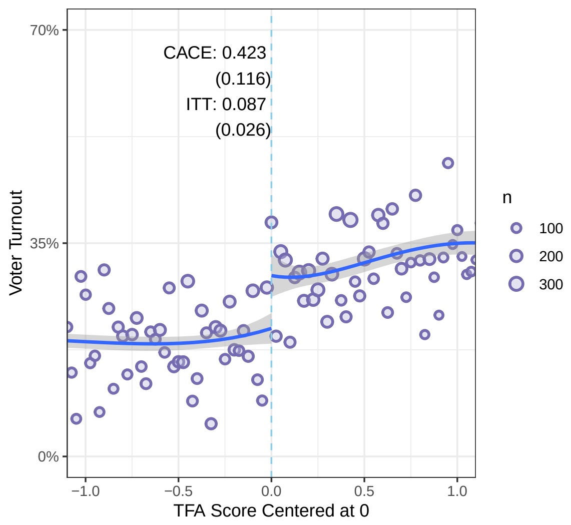

(c) Post-TFA, Match 1 (2010-2014)

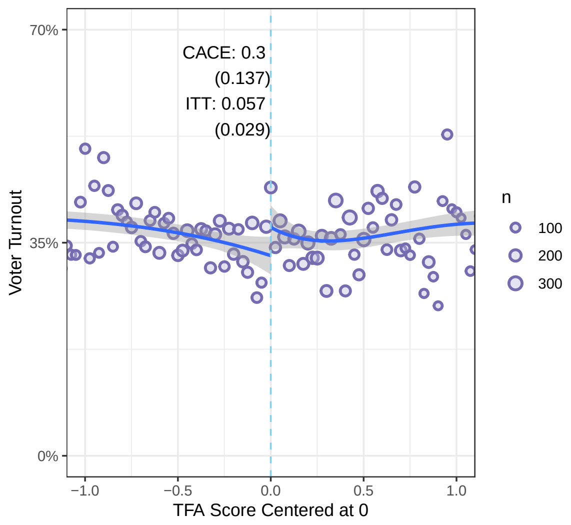

(d) Post-TFA, Match 2 (2010-2014)

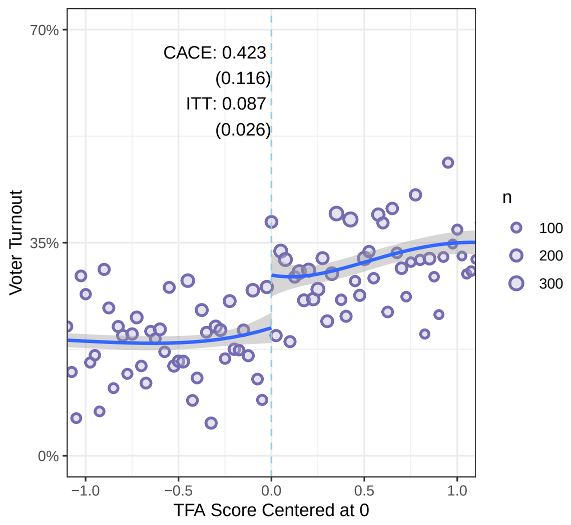

Note: Points to the right of 0 are those who were above the admittance threshold, whereas those to the left are those that are below it. Each sub-figure reports the complier average causal effect (i.e. the CACE) and the intention to treat effect (i.e. the ITT) annotated above the TFA admittance threshold. Therein, the standard errors are in parentheses below the coefficient estimates. The figures show the average levels of voter turnout by selection score. Lines are 4th degree polynomials fitted separately on either side of the cutpoint (*6*). Binned values are sized to indicate number of observations.

Fig. S.14: Effect of TFA Experience on Turnout (Completed Program as Treatment)

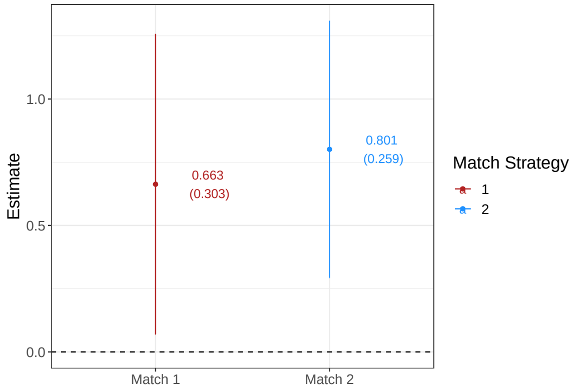

Note: Points represent effect estimates for completion of the TFA program, instrumented by applicants' scores.

A.10 Proposed Congressional Legislation on National Service

Table S.2 shows a sample of legislation proposed in Congress over the past 13 Congresses that has to do with national service.[2] These do not include all bills that have been introduced nor do they include Congressional resolutions, committee reports, committee publications, or other parts of the Congressional Record.

[2]Specifically, these are all the bills that mention "AmeriCorps," which is the federally funded national service organization, where participants provide service domestically. Teach For America is an AmeriCorps program.

Table S.2: Recent Legislation on National Service

Legislation Number	Congress	Title	Sponsor	Introduced	Cosponsors
H.R. 3941	117	Segal AmeriCorps Education Award Tax Relief Act of 2021	Rep. Larson, John B. [D-CT-1]	6/16/21	1
S. 2075	117	Segal AmeriCorps Education Award Tax Relief Act of 2021	Sen. Bennet, Michael F. [D-CO]	6/16/21	3
S. 1057	117	Civilian Climate Corps Act of 2021	Sen. Coons, Christopher A. [D-DE]	3/25/21	3
H.R. 2241	117	Civilian Climate Corps Act of 2021	Rep. Neguse, Joe [D-CO-2]	3/26/21	2
S. 1165	117	Cultivating Opportunity and Recovery from the Pandemic through Service	Sen. Coons, Christopher A. [D-DE]	4/15/21	16
H.R. 4100	117	CORPS Act	Rep. Price, David E. [D-NC-4]	6/23/21	36
H.R. 932	117	To require the Secretary of Agriculture to conduct a study on national	Rep. Griffith, H. Morgan [R-VA-9]	2/8/21	0
S. 486	117	Rebuild Rural America Act of 2021	Sen. Gillibrand, Kirsten E. [D-NY]	2/25/21	4
H.R. 2361	117	Rebuild Rural America Act of 2021	Rep. Delgado, Antonio [D-NY-19]	4/5/21	15
S. 1244	117	Civilian Climate Corps for Jobs and Justice Act	Sen. Markey, Edward J. [D-MA]	4/20/21	6
H.R. 2670	117	The Civilian Climate Corps for Jobs and Justice Act	Rep. Ocasio-Cortez, Alexandria [D-NY-14]	4/20/21	21
S. 1928	117	National Climate Service Corps and Careers Network Act of 2021	Sen. Cortez Masto, Catherine [D-NV]	5/27/21	0
H.R. 4220	117	National Climate Service Corps and Careers Network Act of 2021	Rep. Chu, Judy [D-CA-27]	6/29/21	7
S. 1181	117	HOPE Act of 2021	Sen. Gillibrand, Kirsten E. [D-NY]	4/15/21	0
H.R. 2336	117	HOPE Act of 2021	Rep. Morelle, Joseph D. [D-NY-25]	4/1/21	3
S. 32	117	Health Force, Resilience Force, and Jobs To Fight COVID-19 Act of 20	Sen. Gillibrand, Kirsten E. [D-NY]	1/22/21	15
H.R. 460	117	Health Force, Resilience Force, and Jobs To Fight COVID-19 Act of 20	Rep. Crow, Jason [D-CO-6]	1/25/21	24
S. 1874	117	Recreation Not Red Tape Act	Sen. Wyden, Ron [D-OR]	5/27/21	0
H.R. 3527	117	Recreation Not Red Tape Act	Rep. Moore, Blake D. [R-UT-1]	5/25/21	3
S. 2244	117	PREP Act of 2021	Sen. Kaine, Tim [D-VA]	6/24/21	1
H.R. 3000	117	Inspire to Serve Act of 2021	Rep. Panetta, Jimmy [D-CA-20]	5/4/21	9
H.R. 1319	117	American Rescue Plan Act of 2021	Rep. Yarmuth, John A. [D-KY-3]	2/24/21	0
H.R. 4502	117	Labor, Health and Human Services, Education, Agriculture, Rural Develo	Rep. DeLauro, Rosa L. [D-CT-3]	7/19/21	0
H.R. 1794	116	Segal AmeriCorps Education Award Tax Relief Act of 2019	Rep. Lewis, John [D-GA-5]	3/14/19	18
S. 1355	116	Segal AmeriCorps Education Award Tax Relief Act of 2019	Sen. Bennet, Michael F. [D-CO]	5/7/19	11
H.R. 5829	116	The National Service GI Bill Act	Rep. Waltz, Michael [R-FL-6]	2/10/20	16
H.R. 6560	116	UNITE Act of 2020	Rep. Garamendi, John [D-CA-3]	4/21/20	24
H.R. 5618	116	Promoting National Service and Reducing Unemployment Act	Del. Norton, Eleanor Holmes [D-DC-At Large]	1/15/20	0
H.R. 6853	116	UNITE Act of 2020	Rep. Phillips, Dean [D-MN-3]	5/13/20	10
S. 3642	116	UNITE Act of 2020	Sen. Markey, Edward J. [D-MA]	5/7/20	2
S. 3964	116	CORPS Act	Sen. Coons, Christopher A. [D-DE]	6/16/20	17
S. 3624	116	Pandemic Response and Opportunity Through National Service Act	Sen. Coons, Christopher A. [D-DE]	5/6/20	26
H.R. 6702	116	Pandemic Response and Opportunity Through National Service Act	Rep. Price, David E. [D-NC-4]	5/5/20	67
S. 1770	116	RISE from Trauma Act	Sen. Durbin, Richard J. [D-IL]	6/10/19	3
H.R. 3180	116	RISE from Trauma Act	Rep. Davis, Danny K. [D-IL-7]	6/10/19	13
S. 3454	116	Educational Award Parity Act	Sen. Coons, Christopher A. [D-DE]	3/12/20	3
H.R. 6251	116	Educational Award Parity Act	Rep. Matsui, Doris O. [D-CA-6]	3/12/20	2
H.R. 5067	116	TEAMS Act	Rep. Meadows, Mark [R-NC-11]	11/13/19	1
S. 2704	116	Rebuild Rural America Act of 2019	Sen. Gillibrand, Kirsten E. [D-NY]	10/24/19	2
H.R. 4874	116	Rebuild Rural America Act of 2019	Rep. Delgado, Antonio [D-NY-19]	10/28/19	9
H.R. 3749	116	Legal Services for Homeless Veterans Act	Rep. Panetta, Jimmy [D-CA-20]	7/12/19	43
H.R. 5176	116	Climate Resiliency Service Corps Act of 2019	Rep. Chu, Judy [D-CA-27]	11/19/19	15
H.R. 6833	116	Utilizing and Supporting Evacuated Peace Corps Volunteers Act	Rep. Phillips, Dean [D-MN-3]	5/12/20	24
S. 2882	116	Wildfire Defense Act	Sen. Harris, Kamala D. [D-CA]	11/18/19	4
H.R. 5091	116	Wildfire Defense Act	Rep. Huffman, Jared [D-CA-2]	11/14/19	20
S. 4302	116	VICTORY Act	Sen. Young, Todd [R-IN]	7/23/20	1
S. 3484	116	HOPE Act of 2020	Sen. Gillibrand, Kirsten E. [D-NY]	3/12/20	0
H.R. 6217	116	HOPE Act of 2020	Rep. Morelle, Joseph D. [D-NY-25]	3/12/20	2
H.R. 6808	116	Health Force and Resilience Force Act of 2020	Rep. Crow, Jason [D-CO-6]	5/12/20	20
S. 3606	116	Health Force and Resilience Force Act of 2020	Sen. Gillibrand, Kirsten E. [D-NY]	5/5/20	13
H.R. 1470	116	Anti-Hunger Empowerment Act of 2019	Rep. Serrano, Jose E. [D-NY-15]	2/28/19	11
H.R. 841	116	People CARE Act	Rep. Davidson, Warren [R-OH-8]	1/29/19	0
S. 1967	116	Recreation Not Red Tape Act	Sen. Wyden, Ron [D-OR]	6/25/19	4
H.R. 3458	116	Recreation Not Red Tape Act	Rep. Bishop, Rob [R-UT-1]	6/25/19	6
H.R. 7591	116	Fostering Healthy Transitions into Adulthood Act of 2020	Rep. Lewis, John [D-GA-5]	7/13/20	0
H.R. 3879	116	SOAR Act	Rep. Haaland, Debra A. [D-NM-1]	7/23/19	27
S. 752	116	PREP Act of 2019	Sen. Kaine, Tim [D-VA]	3/12/19	3
H.R. 2065	116	Affordable Loans for Any Student Act	Rep. DeLauro, Rosa L. [D-CT-3]	4/3/19	0
S. 1002	116	Affordable Loans for Any Student Act	Sen. Merkley, Jeff [D-OR]	4/3/19	10
H.R. 7614	116	Departments of Labor, Health and Human Services, and Education, and Re	Rep. DeLauro, Rosa L. [D-CT-3]	7/15/20	0
H.R. 6415	116	Inspire to Serve Act of 2020	Rep. Panetta, Jimmy [D-CA-20]	3/27/20	11
H.R. 7105	116	Johnny Isakson and David P. Roe, M.D. Veterans Health Care and Benefit	Rep. Levin, Mike [D-CA-49]	6/4/20	10
H.R. 2740	116	Labor, Health and Human Services, Education, Defense, State, Foreign O	Rep. DeLauro, Rosa L. [D-CT-3]	5/15/19	0

Legislation Number	Congress	Title	Sponsor	Introduced	Cosponsors
S. 4800	116	The Heroes Act	Sen. Schumer, Charles E. [D-NY]	10/19/20	0
H.R. 8406	116	The Heroes Act	Rep. Lowey, Nita M. [D-NY-17]	9/29/20	10
H.R. 925	116	The Heroes Act	Rep. Thompson, Mike [D-CA-5]	1/30/19	34
H.R. 6800	116	The Heroes Act	Rep. Lowey, Nita M. [D-NY-17]	5/12/20	11
H.R. 7617	116	Defense, Commerce, Justice, Science, Energy and Water Development, Fin	Rep. Visclosky, Peter J. [D-IN-1]	7/16/20	0
H.R. 1865	116	Further Consolidated Appropriations Act, 2020	Rep. Pascrell, Bill, Jr. [D-NJ-9]	3/25/19	304
H.R. 133	116	Consolidated Appropriations Act, 2021	Rep. Cuellar, Henry [D-TX-28]	1/3/19	4
S. 1042	115	Segal AmeriCorps Education Award Enhancement Act of 2017	Sen. Bennet, Michael F. [D-CO]	5/4/17	12
H.R. 1165	115	Segal AmeriCorps Education Award Tax Relief Act of 2017	Rep. Lewis, John [D-GA-5]	2/16/17	0
H.R. 1602	115	Segal AmeriCorps Education Award Tax Relief Act of 2017	Rep. Lewis, John [D-GA-5]	3/17/17	9
H.R. 6373	115	Promoting National Service and Reducing Unemployment Act	Del. Norton, Eleanor Holmes [D-DC-At Large]	7/13/18	0
H.R. 5931	115	To repeal the authority to establish certain special volunteer program	Rep. Biggs, Andy [R-AZ-5]	5/23/18	0
S. 3665	115	21st Century American Service Act	Sen. Duckworth, Tammy [D-IL]	11/28/18	4
H.R. 3140	115	ACTION for National Service Act	Rep. Larson, John B. [D-CT-1]	6/29/17	164
H.R. 7286	115	21st Century American Service Act	Rep. Moulton, Seth [D-MA-6]	12/12/18	0
H.R. 3130	115	INSPIRE Act	Rep. Huffman, Jared [D-CA-2]	6/29/17	1
H.R. 1189	115	Anti-hunger Empowerment Act of 2017	Rep. Serrano, Jose E. [D-NY-15]	2/16/17	1
H.R. 1469	115	Welfare Benefit Reform and Alignment Commission (BRAC) Act	Rep. Davidson, Warren [R-OH-8]	3/9/17	10
S. 1633	115	Recreation Not Red-Tape Act	Sen. Wyden, Ron [D-OR]	7/26/17	4
H.R. 3400	115	Recreation Not Red Tape Act	Rep. Bishop, Rob [R-UT-1]	7/26/17	13
S. 1771	115	Departments of Labor, Health and Human Services, and Education, and Re	Sen. Blunt, Roy [R-MO]	9/7/17	0
S. 1290	115	Welfare Reform and Upward Mobility Act	Sen. Lee, Mike [R-UT]	6/5/17	4
H.R. 2832	115	Welfare Reform and Upward Mobility Act	Rep. Jordan, Jim [R-OH-4]	6/8/17	79
H.R. 3358	115	Departments of Labor, Health and Human Services, and Education, and Re	Rep. Cole, Tom [R-OK-4]	7/24/17	0
S. 3584	115	Affordable Loans for Any Student Act	Sen. Merkley, Jeff [D-OR]	10/11/18	9
S. 3308	115	PREP Act of 2018	Sen. Kaine, Tim [D-VA]	7/31/18	0
S. 3158	115	Departments of Labor, Health and Human Services, and Education, and Re	Sen. Blunt, Roy [R-MO]	6/28/18	0
H.R. 6470	115	Departments of Labor, Health and Human Services, and Education, and Re	Rep. Cole, Tom [R-OK-4]	7/23/18	0
H.R. 6157	115	Department of Defense and Labor, Health and Human Services, and Educat	Rep. Granger, Kay [R-TX-12]	6/20/18	0
H.R. 244	115	Consolidated Appropriations Act, 2017	Rep. Cook, Paul [R-CA-8]	1/4/17	28
H.R. 3354	115	Interior and Environment, Agriculture and Rural Development, Commerce,	Rep. Calvert, Ken [R-CA-42]	7/21/17	0
H.R. 1625	115	Consolidated Appropriations Act, 2018	Rep. Royce, Edward R. [R-CA-39]	3/20/17	40
S. 193	115	Volunteer Income Tax Assistance (VITA) Act	Sen. Brown, Sherrod [D-OH]	1/23/17	1
H.R. 605	115	Volunteer Income Tax Assistance (VITA) Act	Rep. Davis, Danny K. [D-IL-7]	1/23/17	0
H.R. 4949	114	Segal AmeriCorps Education Award Tax Relief Act of 2016	Rep. Lewis, John [D-GA-5]	4/14/16	2
S. 1710	114	AmeriCorps School Turnaround Act of 2015	Sen. Brown, Sherrod [D-OH]	7/7/15	2
H.R. 4821	114	Promoting National Service and Reducing Unemployment Act	Del. Norton, Eleanor Holmes [D-DC-At Large]	3/21/16	0
H.R. 5062	114	21st Century American Service Act	Rep. Duckworth, Tammy [D-IL-8]	4/26/16	2
H.R. 5844	114	ACTION for National Service Act	Rep. Larson, John B. [D-CT-1]	7/14/16	125
H.R. 4121	114	Anti-hunger Empowerment Act of 2015	Rep. Serrano, Jose E. [D-NY-15]	11/19/15	6
S. 3040	114	Departments of Labor, Health and Human Services, and Education, and Re	Sen. Blunt, Roy [R-MO]	6/9/16	0
H.R. 3020	114	Departments of Labor, Health and Human Services, and Education, and Re	Rep. Cole, Tom [R-OK-4]	7/10/15	0
S. 1695	114	Departments of Labor, Health and Human Services, and Education, and Re	Sen. Blunt, Roy [R-MO]	6/25/15	0
S. 3047	114	Welfare Reform and Upward Mobility Act	Sen. Lee, Mike [R-UT]	6/9/16	3
H.R. 5360	114	Welfare Reform and Upward Mobility Act	Rep. Jordan, Jim [R-OH-4]	5/26/16	4
H.R. 5926	114	Departments of Labor, Health and Human Services, and Education, and Re	Rep. Cole, Tom [R-OK-4]	7/22/16	0
S. 2132	114	An Act Making Appropriations to Stop Regulatory Excess and for Other P	Sen. Cochran, Thad [R-MS]	10/5/15	2
H.R. 2029	114	Consolidated Appropriations Act, 2016	Rep. Dent, Charles W. [R-PA-15]	4/24/15	0
H.R. 2737	113	Segal AmeriCorps Education Award Tax Relief Act of 2013	Rep. Lewis, John [D-GA-5]	7/18/13	30
H.R. 4054	113	Promoting National Service and Reducing Unemployment Act	Del. Norton, Eleanor Holmes [D-DC-At Large]	2/11/14	3
H.R. 208	113	Anti-hunger Empowerment Act of 2013	Rep. Serrano, Jose E. [D-NY-15]	1/4/13	4
S. 2015	113	Welfare Reform and Upward Mobility Act	Sen. Lee, Mike [R-UT]	2/11/14	3
S. 1284	113	Departments of Labor, Health and Human Services, and Education, and Re	Sen. Harkin, Tom [D-IA]	7/11/13	0
H.R. 4731	113	Welfare Reform Act of 2014	Rep. Jordan, Jim [R-OH-4]	5/22/14	13
H.R. 5464	113	Departments of Labor, Health and Human Services, and Education, and Re	Rep. DeLauro, Rosa L. [D-CT-3]	9/15/14	4
H.R. 3547	113	Consolidated Appropriations Act, 2014	Rep. Smith, Lamar [R-TX-21]	11/20/13	3
H.R. 83	113	Consolidated and Further Continuing Appropriations Act, 2015	Del. Christensen, Donna M. [D-VI-At Large]	1/3/13	4
H.R. 1816	112	Segal AmeriCorps Education Award Tax Relief Act of 2011	Rep. Lewis, John [D-GA-5]	5/10/11	1
H.R. 3060	112	Economic Growth and Reducing Unemployment Act	Del. Norton, Eleanor Holmes [D-DC-At Large]	9/23/11	2
H.R. 3794	112	Volunteer Freedom Act	Rep. Stutzman, Marlin A. [R-IN-3]	1/18/12	2
H.R. 350	112	Anti-hunger Empowerment Act of 2011	Rep. Serrano, Jose E. [D-NY-16]	1/19/11	11
S. 1599	112	Departments of Labor, Health and Human Services, and Education, and Re	Sen. Harkin, Tom [D-IA]	9/22/11	0
S. 1565	112	National Opportunity and Community Renewal Act	Sen. Casey, Robert P., Jr. [D-PA]	9/15/11	0
H.R. 1135	112	Welfare Reform Act of 2011	Rep. Jordan, Jim [R-OH-4]	3/16/11	4
S. 1904	112	Welfare Reform Act of 2011	Sen. DeMint, Jim [R-SC]	11/17/11	11
H.R. 1167	112	Welfare Reform Act of 2011	Rep. Jordan, Jim [R-OH-4]	3/17/11	75
S. 3295	112	Departments of Labor, Health and Human Services, and Education, and Re	Sen. Harkin, Tom [D-IA]	6/14/12	0
H.R. 3070	112	Departments of Labor, Health and Human Services, and Education, and Re	Rep. Rehberg, Denny [R-MT-At Large]	9/29/11	0
H.R. 3671	112	Consolidated Appropriations Act, 2012	Rep. Rogers, Harold [R-KY-5]	12/14/11	0
H.R. 2055	112	Consolidated Appropriations Act, 2012	Rep. Culberson, John Abney [R-TX-7]	5/31/11	0
H.R. 1596	111	Segal AmeriCorps Education Award Tax Relief Act of 2009	Rep. Lewis, John [D-GA-5]	3/18/09	67
H.R. 1154	111	ACTION Act	Rep. DeLauro, Rosa L. [D-CT-3]	2/24/09	15
S. 464	111	ACTION Act	Sen. Dodd, Christopher J. [D-CT]	2/24/09	9
H.R. 2332	111	Future Physicians Serving America Act of 2009	Rep. McMahon, Michael E. [D-NY-13]	5/7/09	8
H.R. 1388	111	Serve America Act	Rep. McCarthy, Carolyn [D-NY-4]	3/9/09	37
H.R. 1363	111	GothamCorps Authorization Act of 2009	Rep. Weiner, Anthony D. [D-NY-9]	3/5/09	0
S. 277	111	Serve America Act	Sen. Kennedy, Edward M. [D-MA]	1/16/09	42
H.R. 1153	111	Summer of Service Act of 2009	Rep. DeLauro, Rosa L. [D-CT-3]	2/24/09	18

Legislation Number	Congress	Title	Sponsor	Introduced	Cosponsors
S. 466	111	Summer of Service Act of 2009	Sen. Dodd, Christopher J. [D-CT]	2/24/09	7
H.R. 176	111	Anti-hunger Empowerment Act of 2009	Rep. Serrano, Jose E. [D-NY-16]	1/6/09	20
H.R. 3293	111	Departments of Labor, Health and Human Services, and Education, and Re	Rep. Obey, David R. [D-WI-7]	7/22/09	0
H.R. 679	111	American Recovery and Reinvestment Act of 2009	Rep. Obey, David R. [D-WI-7]	1/26/09	0
H.R. 1105	111	Omnibus Appropriations Act, 2009	Rep. Obey, David R. [D-WI-7]	2/23/09	0
S. 336	111	American Recovery and Reinvestment Act of 2009	Sen. Inouye, Daniel K. [D-HI]	1/27/09	0
H.R. 3288	111	Consolidated Appropriations Act, 2010	Rep. Olver, John W. [D-MA-1]	7/22/09	0
H.R. 1	111	American Recovery and Reinvestment Act of 2009	Rep. Obey, David R. [D-WI-7]	1/26/09	9
H.R. 2847	111	Hiring Incentives to Restore Employment Act	Rep. Mollohan, Alan B. [D-WV-1]	6/12/09	0
H.R. 3082	111	Continuing Appropriations and Surface Transportation Extensions Act, 2	Rep. Edwards, Chet [D-TX-17]	6/26/09	0
H.R. 6407	110	Segal AmeriCorps Education Award Tax Relief Act of 2008	Rep. Lewis, John [D-GA-5]	6/26/08	34
S. 3037	110	AmeriCorps: Together Improving Our Nation Act	Sen. Dodd, Christopher J. [D-CT]	5/20/08	13
H.R. 5563	110	Generations Invigorating Volunteerism and Education Act	Rep. McCarthy, Carolyn [D-NY-4]	3/10/08	27
H.R. 2857	110	GIVE Act	Rep. McCarthy, Carolyn [D-NY-4]	6/26/07	32
H.R. 6081	110	Heroes Earnings Assistance and Relief Tax Act of 2008	Rep. Rangel, Charles B. [D-NY-15]	5/16/08	28
H.R. 3997	110	Defenders of Freedom Tax Relief Act of 2007	Rep. Rangel, Charles B. [D-NY-15]	10/30/07	0
H.R. 3043	110	Departments of Labor, Health and Human Services, and Education, and Re	Rep. Obey, David R. [D-WI-7]	7/13/07	0
S. 3230	110	Departments of Labor, Health and Human Services, and Education, and Re	Sen. Harkin, Tom [D-IA]	7/8/08	0
H.R. 2969	110	GothamCorps Authorization Act of 2007	Rep. Weiner, Anthony D. [D-NY-9]	7/10/07	0
S. 1710	110	Departments of Labor, Health and Human Services, and Education, and Re	Sen. Harkin, Tom [D-IA]	6/27/07	0
H.R. 1880	110	Summer of Service Act of 2007	Rep. DeLauro, Rosa L. [D-CT-3]	4/17/07	18
S. 1128	110	Summer of Service Act of 2007	Sen. Dodd, Christopher J. [D-CT]	4/17/07	8
H.R. 7154	110	T-FELAS Act	Rep. Loebsack, David [D-IA-2]	9/26/08	0
S. 778	110	Teaching Fellows for Expanded Learning and After-School Act of 2007	Sen. Kennedy, Edward M. [D-MA]	3/6/07	4
H.R. 206	110	Anti-hunger Empowerment Act of 2007	Rep. Serrano, Jose E. [D-NY-16]	1/4/07	4
H.R. 2669	110	College Cost Reduction and Access Act	Rep. Miller, George [D-CA-7]	6/12/07	31
H.R. 2764	110	Consolidated Appropriations Act, 2008	Rep. Lowey, Nita M. [D-NY-18]	6/18/07	0
H.R. 4137	110	Higher Education Opportunity Act	Rep. Miller, George [D-CA-7]	11/9/07	29
H.R. 3796	109	AmeriCorps Disaster Relief Corps Act of 2005	Rep. Ford, Harold E., Jr. [D-TN-9]	9/15/05	10
H.R. 3958	109	Louisiana Katrina Reconstruction Act	Rep. Melancon, Charlie [D-LA-3]	9/29/05	0
S. 1766	109	Louisiana Katrina Reconstruction Act	Sen. Vitter, David [R-LA]	9/22/05	1
S. 1765	109	Louisiana Katrina Reconstruction Act	Sen. Landrieu, Mary L. [D-LA]	9/22/05	1
H.R. 3709	109	To amend title 10, United States Code, to remove the Peace Corps as an	Rep. Kline, John [R-MN-2]	9/8/05	38
H.R. 6358	109	Summer of Service Act of 2006	Rep. DeLauro, Rosa L. [D-CT-3]	12/5/06	11
S. 4053	109	Summer of Service Act of 2006	Sen. Dodd, Christopher J. [D-CT]	11/15/06	7
H.R. 3010	109	Departments of Labor, Health and Human Services, and Education, and Re	Rep. Regula, Ralph [R-OH-16]	6/21/05	0
H.R. 5647	109	Departments of Labor, Health and Human Services, and Education, and Re	Rep. Regula, Ralph [R-OH-16]	6/20/06	0
S. 3708	109	Departments of Labor, Health and Human Services, and Education, and Re	Sen. Specter, Arlen [R-PA]	7/20/06	0
H.R. 5158	109	Anti-hunger Empowerment Act of 2006	Rep. Serrano, Jose E. [D-NY-16]	4/6/06	9
H.R. 4939	109	Emergency Supplemental Appropriations Act for Defense, the Global War	Rep. Lewis, Jerry [R-CA-41]	3/13/06	0
S. 1042	109	National Defense Authorization Act for Fiscal Year 2006	Sen. Warner, John [R-VA]	5/17/05	0
S. 1043	109	Department of Defense Authorization Act for Fiscal Year 2006	Sen. Warner, John [R-VA]	5/17/05	0
H.R. 1815	109	National Defense Authorization Act for Fiscal Year 2006	Rep. Hunter, Duncan [R-CA-52]	4/26/05	1
H.R. 4459	109	Service for School Act of 2005	Rep. Ford, Harold E., Jr. [D-TN-9]	12/7/05	0
S. 1276	108	Strengthen AmeriCorps Program Act	Sen. Bond, Christopher S. [R-MO]	6/18/03	13
H.R. 2552	108	Strengthen AmeriCorps Program Act	Rep. Van Hollen, Chris [D-MD-8]	6/19/03	0
H.R. 2125	108	Rite of Passage Community Service Act	Rep. DeLauro, Rosa L. [D-CT-3]	5/15/03	48
S. 1274	108	Call to Service Act	Sen. Kennedy, Edward M. [D-MA]	6/17/03	4
S. 1383	108	Legislative Branch Appropriations Act, 2004	Sen. Campbell, Ben Nighthorse [R-CO]	7/9/03	0
S. 1584	108	Departments of Veterans Affairs and Housing and Urban Development, and	Sen. Bond, Christopher S. [R-MO]	9/5/03	0
H.R. 1396	108	Spectrum Commons and Digital Dividends Act of 2003	Rep. Markey, Edward J. [D-MA-7]	3/20/03	2
H.R. 5041	108	Departments of Veterans Affairs and Housing and Urban Development, and	Rep. Walsh, James T. [R-NY-25]	9/9/04	0
S. 2825	108	Departments of Veterans Affairs and Housing and Urban Development, and	Sen. Bond, Christopher S. [R-MO]	9/21/04	0
H.R. 2861	108	Departments of Veterans Affairs and Housing and Urban Development, and	Rep. Walsh, James T. [R-NY-25]	7/24/03	0
H.R. 2657	108	Legislative Branch Appropriations Act, 2004	Rep. Kingston, Jack [R-GA-1]	7/1/03	0
H.R. 2673	108	Consolidated Appropriations Act, 2004	Rep. Bonilla, Henry [R-TX-23]	7/9/03	0
H.R. 743	108	Social Security Protection Act of 2004	Rep. Shaw, E. Clay, Jr. [R-FL-22]	2/12/03	31
H.R. 4818	108	Consolidated Appropriations Act, 2005	Rep. Kolbe, Jim [R-AZ-8]	7/13/04	0
S. 448	108	Leave No Child Behind Act of 2003	Sen. Dodd, Christopher J. [D-CT]	2/26/03	14
H.R. 936	108	Leave No Child Behind Act of 2003	Rep. Miller, George [D-CA-7]	2/26/03	96
S. 2917	108	A bill to amend the National Aeronautics and Space Act of 1958 to esta	Sen. Brownback, Sam [R-KS]	10/7/04	1
S. 1352	107	AmeriCorps Reform and Charitable Expansion Act	Sen. Santorum, Rick [R-PA]	8/3/01	0
S. 1792	107	Call to Service Act of 2001	Sen. Bayh, Evan [D-IN]	12/10/01	6
H.R. 3465	107	Call to Service Act of 2001	Rep. Ford, Harold E., Jr. [D-TN-9]	12/12/01	22
H.R. 1149	107	NET Corps Act	Rep. Honda, Michael M. [D-CA-15]	3/21/01	52
H.R. 4803	107	Right of Passage Community Service Act	Rep. DeLauro, Rosa L. [D-CT-3]	5/22/02	51
H.R. 4641	107	Wireless Technology Investment and Digital Dividends Act of 2002	Rep. Markey, Edward J. [D-MA-7]	5/2/02	2
H.R. 4854	107	Citizen Service Act of 2002	Rep. Hoekstra, Peter [R-MI-2]	5/24/02	31
S. 2515	107	Department of Defense Authorization Act for Fiscal Year 2003	Sen. Levin, Carl [D-MI]	5/15/02	0
S. 1216	107	Departments of Veterans Affairs and Housing and Urban Development, and	Sen. Mikulski, Barbara A. [D-MD]	7/20/01	0
S. 2514	107	National Defense Authorization Act for Fiscal Year 2003	Sen. Levin, Carl [D-MI]	5/15/02	0
S. 2797	107	Departments of Veterans Affairs and Housing and Urban Development, and	Sen. Mikulski, Barbara A. [D-MD]	7/25/02	0
H.R. 2620	107	Departments of Veterans Affairs and Housing and Urban Development, and	Rep. Walsh, James T. [R-NY-25]	7/25/01	0

Legislation Number	Congress	Title	Sponsor	Introduced	Cosponsors
H.R. 4546	107	Bob Stump National Defense Authorization Act for Fiscal Year 2003	Rep. Stump, Bob [R-AZ-3]	4/23/02	1
S. 940	107	Leave No Child Behind Act of 2001	Sen. Dodd, Christopher J. [D-CT]	5/23/01	13
H.R. 1990	107	Leave No Child Behind Act of 2001	Rep. Miller, George [D-CA-7]	5/24/01	94
H.R. 1	107	No Child Left Behind Act of 2001	Rep. Boehner, John A. [R-OH-8]	3/22/01	84
H.R. 878	106	Americorps Program Elimination Act	Rep. Tiahrt, Todd [R-KS-4]	2/25/99	42
H.R. 5425	106	To amend the National and Community Service Act of 1990 to include par	Rep. Weldon, Curt [R-PA-7]	10/6/00	0
S. 1292	106	Department of the Interior and Related Agencies Appropriations Act, 20	Sen. Gorton, Slade [R-WA]	6/28/99	0
H.R. 2466	106	Department of the Interior and Related Agencies Appropriations Act, 20	Rep. Regula, Ralph [R-OH-16]	7/2/99	0
H.R. 974	106	District of Columbia College Access Act of 1999	Rep. Davis, Tom [R-VA-11]	3/4/99	13
S. 1465	106	Safe Schools Act of 1999	Sen. Lincoln, Blanche L. [D-AR]	7/29/99	2
H.R. 4578	106	Department of the Interior and Related Agencies Appropriations Act, 20	Rep. Regula, Ralph [R-OH-16]	6/1/00	0
H.R. 3423	106	Department of the Interior and Related Agencies Appropriations Act, 20	Rep. Young, C. W. Bill [R-FL-10]	11/17/99	0
S. 1596	106	Departments of Veterans Affairs and Housing and Urban Development, and	Sen. Bond, Christopher S. [R-MO]	9/16/99	0
H.R. 5482	106	Departments of Veterans Affairs and Housing and Urban Development, and	Rep. Walsh, James T. [R-NY-25]	10/18/00	0
H.R. 2684	106	Departments of Veterans Affairs and Housing and Urban Development, and	Rep. Walsh, James T. [R-NY-25]	8/3/99	0
H.R. 4635	106	Department of Veterans Affairs and Housing and Urban Development, and	Rep. Walsh, James T. [R-NY-25]	6/12/00	0
H.R. 3194	106	Consolidated Appropriations Act, 2000	Rep. Istook, Ernest J., Jr. [R-OK-5]	11/2/99	0
H.R. 993	105	Americorps Program Elimination Act	Rep. Tiahrt, Todd [R-KS-4]	3/6/97	65
H.R. 3440	105	Alternative Routes to Teacher Certification Act of 1998	Rep. Roemer, Tim [D-IN-3]	3/11/98	5
H.R. 4193	105	Department of the Interior and Related Agencies Appropriations Act, 19	Rep. Regula, Ralph [R-OH-16]	7/8/98	0
H.R. 2107	105	Department of the Interior and Related Agencies Appropriations Act, 19	Rep. Regula, Ralph [R-OH-16]	7/1/97	0
H.R. 2852	105	New Century Teachers Act	Rep. Kildee, Dale E. [D-MI-9]	11/6/97	1
S. 1209	105	A bill improving teacher preparation and recruitment.	Sen. Kennedy, Edward M. [D-MA]	9/23/97	2
S. 2237	105	Department of the Interior and Related Agencies Appropriations Act, 19	Sen. Gorton, Slade [R-WA]	6/26/98	0
S. 1034	105	Departments of Veterans Affairs and Housing and Urban Development, and	Sen. Bond, Christopher S. [R-MO]	7/17/97	0
H.R. 2158	105	Departments of Veterans Affairs and Housing and Urban Development, and	Rep. Lewis, Jerry [R-CA-40]	7/11/97	0
S. 2168	105	Departments of Veterans Affairs and Housing and Urban Development, and	Sen. Bond, Christopher S. [R-MO]	6/12/98	0
S. 1882	105	Higher Education Amendments of 1998	Sen. Jeffords, James M. [R-VT]	3/30/98	5
H.R. 4328	105	Omnibus Consolidated and Emergency Supplemental Appropriations Act, 19	Rep. Wolf, Frank R. [R-VA-10]	7/24/98	0
H.R. 4194	105	Departments of Veterans Affairs and Housing and Urban Development, and	Rep. Lewis, Jerry [R-CA-40]	7/8/98	0
H.R. 6	105	Higher Education Amendments of 1998	Rep. McKeon, Howard P. "Buck"" [R-CA-25]"	1/7/97	13
H.R. 3662	104	Department of the Interior and Related Agencies Appropriations Act, 19	Rep. Regula, Ralph [R-OH-16]	6/18/96	0
H.R. 1977	104	Department of the Interior and Related Agencies Appropriations Act, 19	Rep. Regula, Ralph [R-OH-16]	6/30/95	0
H.R. 3019	104	Omnibus Consolidated Rescissions and Appropriations Act of 1996	Rep. Livingston, Bob [R-LA-1]	3/5/96	0
S. 1594	104	An original bill making omnibus consolidated rescissions and appropria	Sen. Hatfield, Mark O. [R-OR]	3/6/96	0
H.R. 3666	104	Departments of Veterans Affairs and Housing and Urban Development, and	Rep. Lewis, Jerry [R-CA-40]	6/18/96	0
H.R. 4278	104	Omnibus Consolidated Appropriations Act, 1997	Rep. Livingston, Bob [R-LA-1]	9/28/96	0
H.R. 3610	104	Omnibus Consolidated Appropriations Act, 1997	Rep. Young, C. W. Bill [R-FL-10]	6/11/96	0

B Supplementary Text

B.1 The Many Mechanisms by Which TFA May Increase Voter Turnout

There are many mechanisms by which TFA may produce the effects outlined in the paper. In this paper, it is not our goal to adjudicate which of these mechanisms are most likely, as 1.) our primary goal is to answer the first-order question of whether experience with TFA causes an increase in voter turnout; and 2.) causal mechanism testing is inherently difficult, if not impossible (*7, 8*). This second point is especially true when testing the effects of larger, more immersive programs. That all being said, here we articulate some of the theoretical reasons why experiences with TFA may combine to produce a sizable increase in voter turnout. Note that the list below is not meant to be comprehensive, but, rather, to illuminate that there are many reasons to expect TFA experience impacts voting.

The first reason why TFA may increase voter turnout rates of its teachers is that it may help participants see the need for public policies to address inequality. TFA participants are placed in disadvantaged communities and are intimately exposed to various social maladies and inequities. TFA participants may look to government institutions as a forum for addressing social ills (*1*), especially since their placement involves working in the public sector as TFA participants are public school educators. Second, TFA may help participants build skills and beliefs that empower them to engage in politics. As hands-on instructors, TFA participants learn social and self-regulator skills and the ability to work with people from a variety of backgrounds, all of which may promote voting (*9–11*). Third, TFA may build social connections—with one's fellow teachers, the TFA network, students, and parents/community leaders—which encourage political action. Stronger social ties may foster an enhanced sense of civic duty to be involved in various forms of community and political engagement (*12*). Fourth, for teachers that remain in teaching, former TFA teachers have a direct stake in educational policy outcomes and may engage to preserve that stake. We note that we can rule out systematic efforts to get out the vote on the part of the national service organization as a mechanism for increasing political participation. As "engaging in activities designed to influence the outcome of an election to Federal office or the outcome of an election to a State or local public office" is prohibited actively for AmeriCorps programs under Section 1310 of the Serve America Act, TFA and other AmeriCorps programs specifically do *not* do anything to encourage participants to register to vote or vote in an election (*13*).

Of note, the fact that the TFA treatment involves being a classroom teacher, may be driving some of the observed effects. While we cannot establish or rule out whether teaching drives our effects, extant research on teacher voter turnout suggests that our observed effects are not being fully driven by teaching (and being mobilized by teacher's unions and large teacher associations like the National Education Association). However, it is plausible that our effects are larger than effects we may observe from a national service program that does not involve teaching in the classroom. Extant research on voter turnout shows that while teachers vote at higher rates than average citizens (*14, 15*), it is not clear that being a teacher causes individuals to vote at higher

rates. For example, those who choose to become teachers may also be more public spirited, and hence, more politically active (*15*). Consonant with this point, recent research has shown that past research arguing that public sector employees, which includes many teachers as teachers are 18 percent of the state and local government workforce (*16*), vote at higher rates have major selection bias problems (*17*). This work finds that when you address omitted variable bias, you do not see evidence that being a government employee causes greater voter turnout. With that said, when we examine studies of just government employees who are educators, there is some suggestive evidence that teaching can cause an increase in voter turnout stemming from occupational self-interest and/or union mobilization. Examining five school districts in California in 1999, previous research finds that teachers who work and live in the same district turnout for local elections with school board races vote at higher levels than teachers who live in a different district than the district in which they work (*15, 18*). However, there is no clear evidence that teachers who do not live in the same district in which they work, and hence, do not have an occupational stake in their local school board elections and are less likely to be mobilized by the unions to vote, turnout at the ballot box at higher rates than the average citizen (*15, 18*). In other words, it is not clear that teaching uniformly causes greater voter turnout.

Though our data does not allow for a well-identified test of teaching alone on turnout, we can look at turnout among TFA applicants who were not admitted to the program and compare those who nonetheless became school teachers to those who did not. Non-admitted applicants who became teachers were *not* significantly more likely to vote than applicants who chose other professions, controlling for birth year, gender, race, and receipt of a Pell Grant (see the first column of Table S.3). This suggests that, within the population of college students who were interested enough in teaching to apply to TFA, those who chose to become a teacher outside the program did not participate in politics at higher rates than those who did not.

Broadening our focus somewhat, we can look at voter turnout rates among young people who do and do not work in education in the general population. We use the 2012 CCES to compare turnout in the 2012 election among people who work in education to those who do not, controlling for gender, age, and race. We limit the sample to respondents born after 1985 with four-year college degrees, to make the results more comparable to the TFA sample analyzed above. As in the TFA analyses, young people who become teachers are not more likely to vote than thoes in other professions. Taken together, these results suggest that teaching alone is unlikely to produce increases in turnout of the magnitude we observe among TFA participants (see the second column of Table S.3). However, these results are only suggestive, and future work should pursue well-identified designs to understand the causal effect of becoming a teacher.

On the flip side, there are several reasons to expect that TFA may actually demobilize its participants. First, TFA participants are highly mobile. Being uprooted from one's social community may be disruptive enough to decrease rates of voting (*19,20*). Second, some have argued that TFA could make participants less trustful of U.S. institutions and, as such, less engaged in politics (*21*). However, theoretically it is somewhat unclear whether decreased trust in this context would actually mobilize TFA participants by making them want to work to change the

Table S.3: Comparing Teachers and Non-Teachers

	TFA Non-Admits	CCES
(Intercept)	10.300	9.131
	(1.508)	(32.132)
Teacher/Education Sector	0.022	0.004
	(0.014)	(0.071)
Birth Year	-0.005	-0.004
	(0.001)	(0.016)
Male (vs. female omitted cat.)	0.048	0.018
	(0.014)	(0.055)
Black (vs. white omitted cat.)	0.015	-0.118
	(0.019)	(0.084)
Hispanic	0.019	-0.149
	(0.023)	(0.078)
Asian	-0.093	-0.189
	(0.026)	(0.094)
Native	0.006	-0.195
	(0.090)	(0.241)
Mixed	-0.003	-0.074
	(0.027)	(0.151)
Other	0.040	0.001
	(0.045)	(0.248)
Received Pell Grant	-0.004	
	(0.014)	
Num.Obs.	5863	825
R2	0.014	0.017
R2 Adj.	0.012	0.006
Std.Errors	HC2	HC2

Notes: Standard errors are in parentheses.

governmental systems in which they work. Ultimately, which of these mechanisms "win out" is an empirical question—one that we explore in this paper.

B.2 Careers of Non-Admits

Survey respondents were asked the following question: "We will now ask you about the last three jobs you have held since 2007. For each position, what is your job title, sector, and start and end date for each of these positions?" Figures that break down the share of non-admits in each job sector are provided below.

Fig. S.15: Sector of First Job Held Since 2007 of Non-Participants

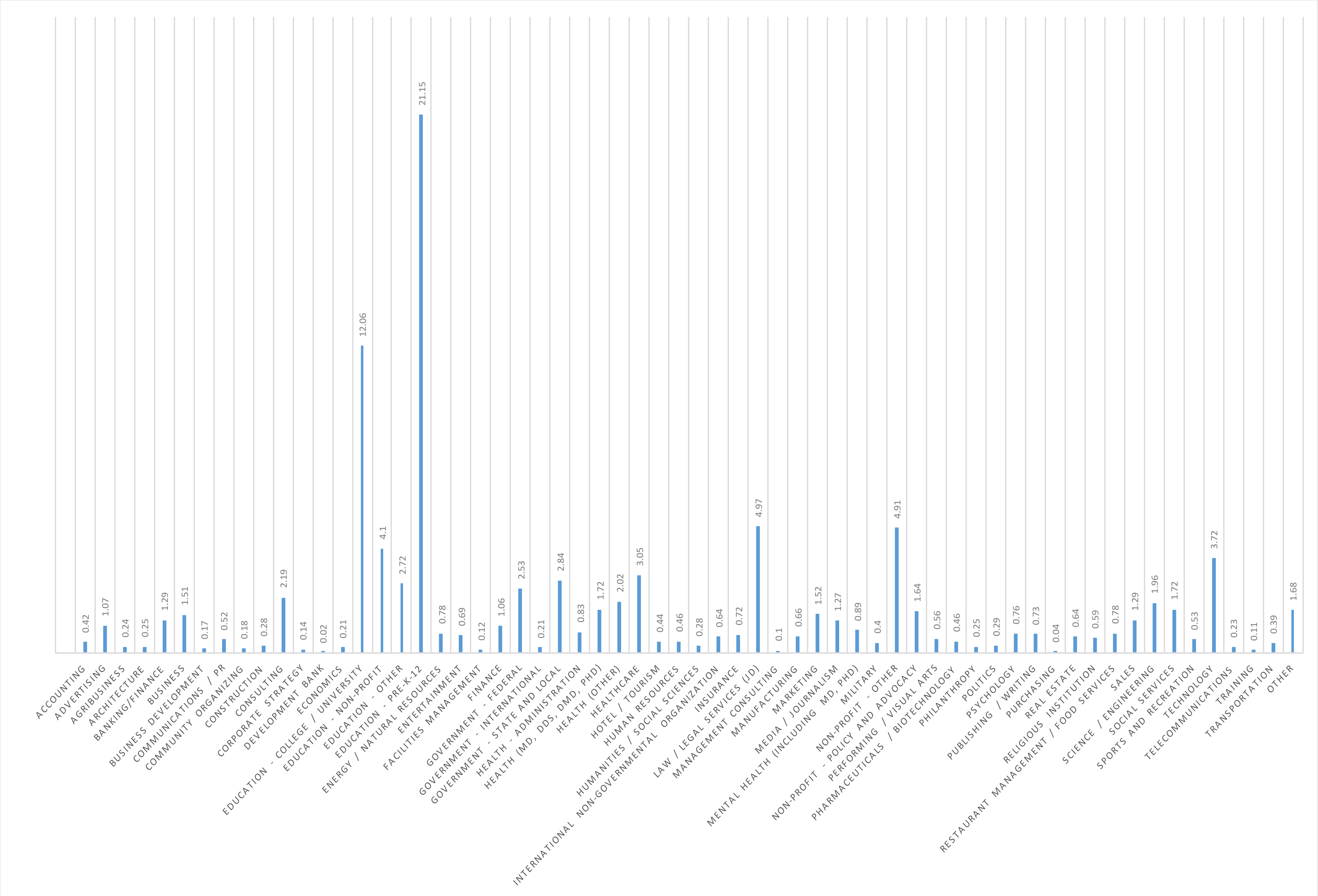

Notes: Survey respondents were asked about the last three jobs they have held since 2007, as that is the first cohort year in our study. This figure displays the percentage of non-participant respondents in each job sector for their first job.

Fig. S.16: Sector of Second Job Held Since 2007 of Non-Participants

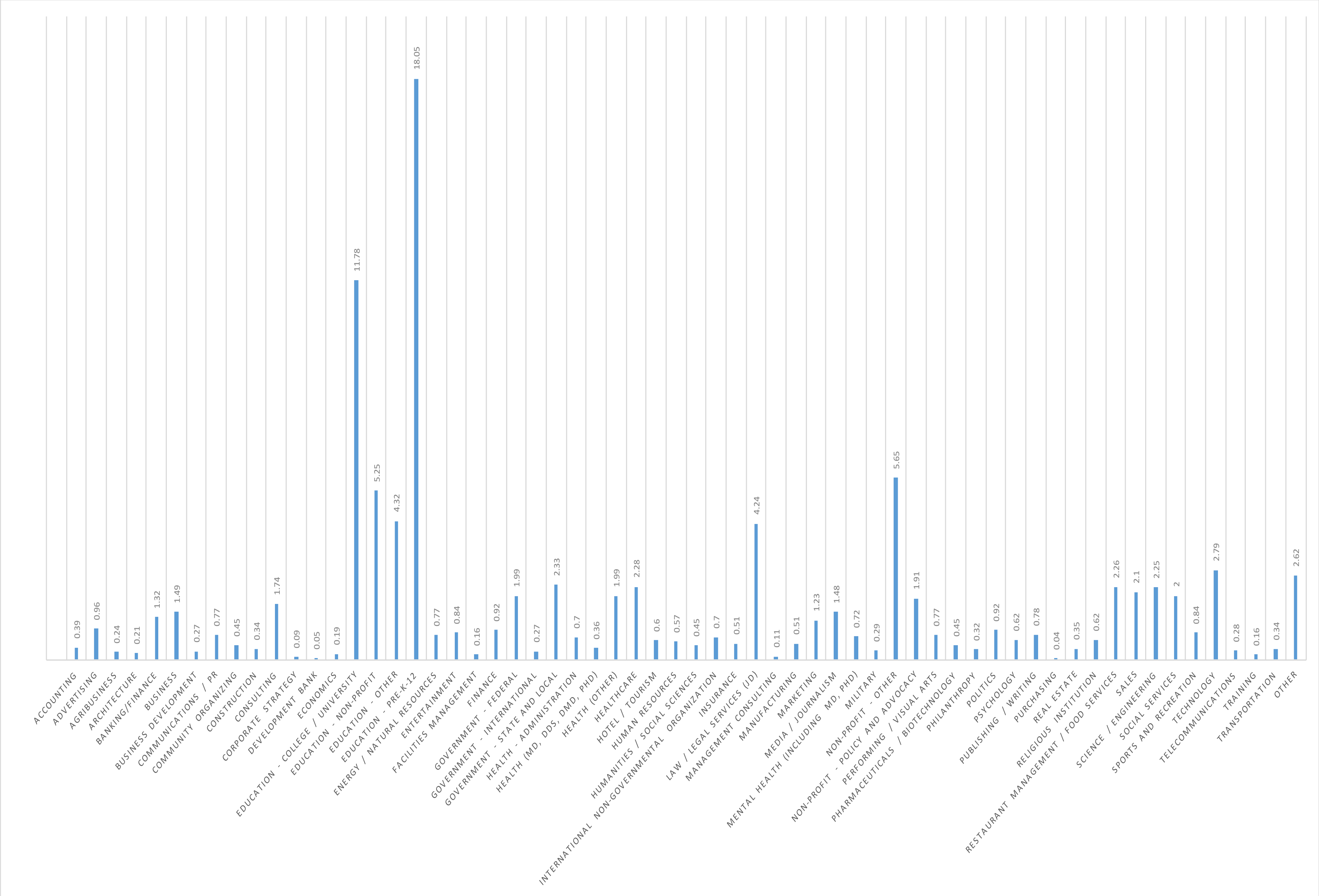

Notes: Survey respondents were asked about the last three jobs they have held since 2007. This figure displays the percentage of non-participant respondents in each job sector for their second job.

Fig. S.17: Sector of Third Job Held Since 2007 of Non-Participants

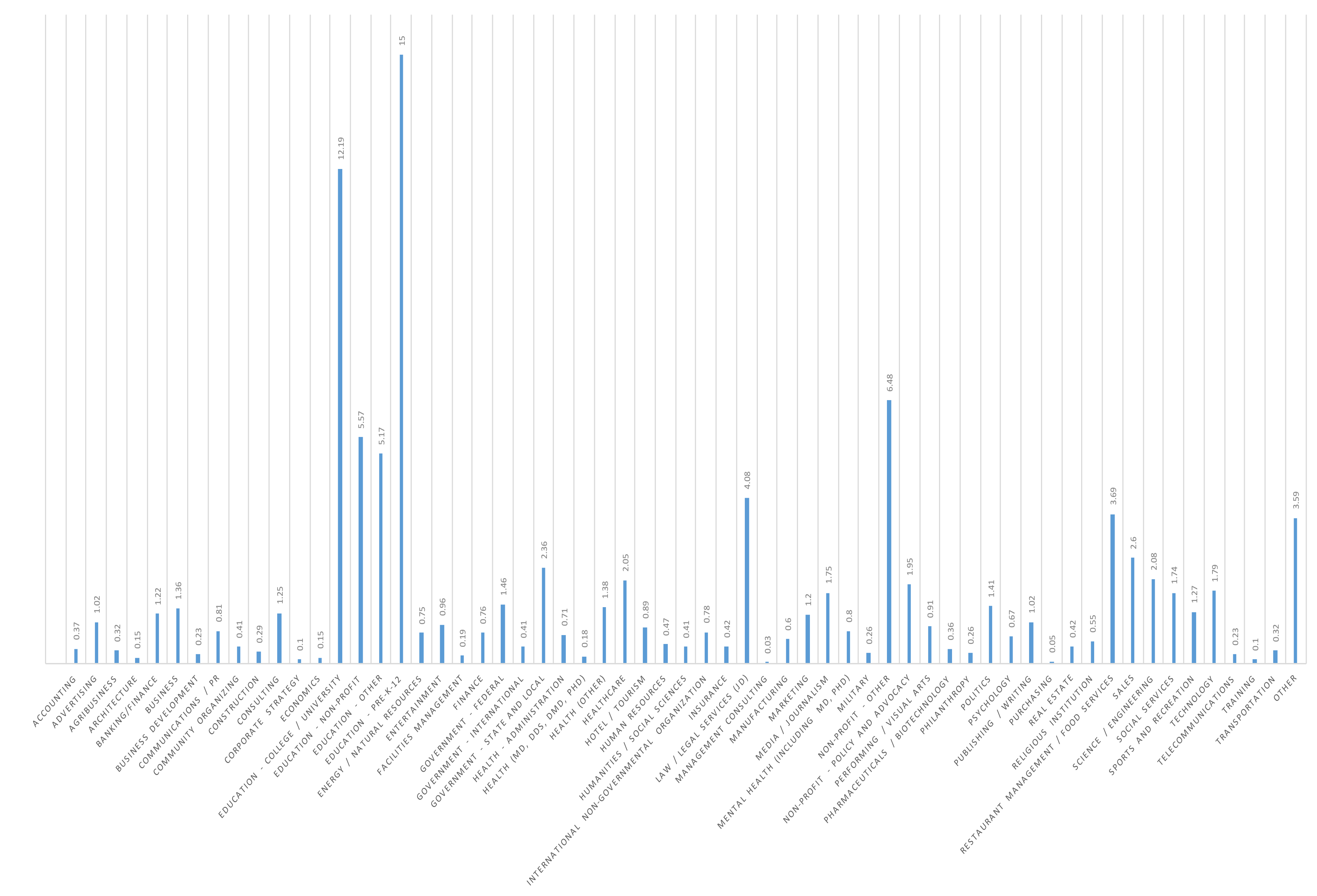

Notes: Survey respondents were asked about the last three jobs they have held since 2007. This figure displays the percentage of non-participant respondents in each job sector for their third job.

Supplemental Materials References

1. CH Mo, KM Conn, When do the advantaged see the disadvantages of others? a quasi-experimental study of national service. **112**, 721–741 (2018).

2. MD Cattaneo, M Jansson, X Ma, Simple Local Polynomial Density Estimators. *J. Am. Stat. Assoc.* **115**, 1449–1455 (2020).

3. T Enamorado, B Fifield, K Imai, Using a probabilistic model to assist merging of large-scale administrative records. **113**, 353–371 (year?).

4. T Enamorado, B Fifield, K Imai, Using a probabilistic model to assist merging of large-scale administrative records. **113**, 353–371 (2019).

5. S Calonico, MD Cattaneo, R Titiunik, Robust nonparametric confidence intervals for regression-discontinuity designs. *Econometrica* **82**, 2295–2326 (2014).

6. S Calonico, MD Cattaneo, R Titiunik, Optimal data-driven regression discontinuity plots. *J. Am. Stat. Assoc.* **110**, 1753–1769 (2015).

7. DP Green, SE Ha, JG Bullock, Enough already about "black box" experiments: Studying mediation is more difficult than most scholars suppose. *Ann. Am. Acad. Polilt. SS* **628**, 200–208 (2010).

8. JG Bullock, DP Green, SE Ha, Yes, but what's the mechanism?(don't expect an easy answer). *J. Pers. Soc. Psychol.* **98**, 550 (2010).

9. JB Holbein, Childhood skill development and adult political participation. **111**, 572–583 (2017).

10. CH Mo, KM Conn, G Anderson-Nilsson, Youth national service and women's political ambition: The case of teach for america. *Politics, Groups, and Identities* **7**, 864–877 (2019).

11. JB Holbein, DS Hillygus, *Making Young Voters: Converting Civic Attitudes Into Civic Action*. (Cambridge University Press), (2020).

12. SJ Rosenstone, JM Hansen, *Mobilization, Participation, and Democracy in America*. (Longman Publishing Group), (1993).

13. The Edward M. Kennedy Serve America Act, (42 U.S.C. § 12501. 2009) (year?).

14. RE Wolfinger, SJ Rosenstone, *Who Votes?* (Brookings Institution Press), (2011).

15. TM Moe, *Special Interest: Teachers Unions and America's Public Schools*. (Brookings Institution Press), (2011).

16. NCES, Characteristics of public school teachers (https://nces.ed.gov/programs/coe/indicator/clrf (Accessed August 9, 2021)) (2021).

17. M Bednarczuk, Voter turnout and bureaucrats across time: A further examination of the bureau voting model in the united states. *Public Admin. Quart.* **41**, 386–414 (2017).

18. TM Moe, The union label on the ballot box. *Education Next* **6**, 58–66 (2006).

19. C Gay, Moving to opportunity: The political effects of a housing mobility experiment. **48**, 147–179 (2012).

20. S Ansolabehere, E Hersh, K Shepsle, , et al., Movers, stayers, and registration: Why age is correlated with registration in the us. **7**, 333–363 (2012).

21. D McAdam, C Brandt, Assessing the effects of voluntary youth service: The case of teach for america. *Soc. Forces* **88**, 945–969 (2009).
